# Molecular and Cellular Response of the Myocardium (H9C2 Cells) Towards Hypoxia and HIF-1α Inhibition

**DOI:** 10.3389/fcvm.2022.711421

**Published:** 2022-07-19

**Authors:** Hari Prasad Osuru, Matthew Lavallee, Robert H. Thiele

**Affiliations:** Thiele Laboratory, Department of Anesthesiology, University of Virginia School of Medicine, Charlottesville, VA, United States

**Keywords:** oxidative stress, intracellular calcium, hypoxia inducible factor, Beclin-1, H9c2 cells, hypoxia and HIF-1**α** inhibition

## Abstract

**Introduction:**

Oxidative phosphorylation is an essential feature of Animalian life. Multiple adaptations have developed to protect against hypoxia, including hypoxia-inducible-factors (HIFs). The major role of HIFs may be in protecting against oxidative stress, not the preservation of high-energy phosphates. The precise mechanism(s) of HIF protection is not completely understood.

**Materials and Methods:**

To better understand the role of hypoxia-inducible-factor-1, we exposed heart/myocardium cells (H9c2) to both normoxia and hypoxia, as well as cobalt chloride (prolyl hydroxylase inhibitor), echniomycin (HIF inhibitor), A2P (anti-oxidant), and small interfering RNA to beclin-1. We measured cell viability, intracellular calcium and adenosine triphosphate, NADP/NADPH ratios, total intracellular reactive oxidative species levels, and markers of oxidative and antioxidant levels measured.

**Results:**

Hypoxia (1%) leads to increased intracellular Ca2+ levels, and this response was inhibited by A2P and echinomycin (ECM). Exposure of H9c2 cells to hypoxia also led to an increase in both mRNA and protein expression for Cav 1.2 and Cav 1.3. Exposure of H9c2 cells to hypoxia led to a decrease in intracellular ATP levels and a sharp reduction in total ROS, SOD, and CAT levels. The impact of hypoxia on ROS was reversed with HIF-1 inhibition through ECM. Exposure of H9c2 cells to hypoxia led to an increase in Hif1a, VEGF and EPO protein expression, as well as a decrease in mitochondrial DNA. Both A2P and ECM attenuated this response to varying degrees.

**Conclusion:**

Hypoxia leads to increased intracellular Ca2+, and inhibition of HIF-1 attenuates the increase in intracellular Ca2+ that occurs with hypoxia. HIF-1 expression leads to decreased adenosine triphosphate levels, but the role of HIF-1 on the production of reactive oxidative species remains uncertain. Anti-oxidants decrease HIF-1 expression in the setting of hypoxia and attenuate the increase in Ca2+ that occurs during hypoxia (with no effect during normoxia). Beclin-1 appears to drive autophagy in the setting of hypoxia (through ATG5) but not in normoxia. Additionally, Beclin-1 is a powerful driver of reactive oxidative species production and plays a role in ATP production. HIF-1 inhibition does not affect autophagy in the setting of hypoxia, suggesting that there are other drivers of autophagy that impact beclin-1.

## Introduction

At the most fundamental level, life can be thought of as a continual, creative process that generates tiny pockets of order in a universe that is growing, in aggregate, increasingly disordered. Because the natural tendency of things is to become disordered, maintaining the structures necessary to sustain life requires the constant influx of energy. In the *Animalian* world with which we concern ourselves, the most efficient source of energy is mitochondrial oxidative phosphorylation (1), in which reducing equivalents (electrons) are donated to the electron transport chain (ETC) where they create a proton gradient that drives ATP synthase and are ultimately accepted by oxygen (2). The presence of oxygen is therefore essential for efficient energy production, and deviations in this finely tuned process (both inadequate and excess oxygenation) can lead to both alterations in ETC function, loss of electrons, and production of free radicals which themselves can cause cellular damage (oxidative stress) in addition to decreasing ATP availability (3). Additionally, because the maintenance of transmembrane ion potentials and neurotransmitter reuptake are ATP-dependent processes, loss of ATP leads to excitotoxicity and ion shifts that ultimately increase intracellular calcium [Ca2+]_*i*_ and lead to cell death (4, 5).

Given the critical role of oxygen as an “electron sink” during oxidative phosphorylation, the ability to adapt to perturbations in oxygen availability is essential to survival. In order to protect against hypoxia, multiple adaptive mechanisms have developed over time, most notably through the hypoxia-inducible factors (HIF) but also through NF-κβ, the unfolded protein response, and mammalian target of rapamycin (mTOR) kinase (6–8). During normoxia, the alpha subunits of HIF proteins (there are three) are continuously degraded by prolyl hydroxylases (PHDs) (3, 9, 10). When intracellular oxygen drops below 10 mm Hg (11), PHDs become ineffective, allowing HIFs to accumulate and activate approximately 1,500 target genes [e.g., vascular endothelial growth factor (VEGF), erythropoietin (EPO), inducible nitric oxide synthase (iNOS), and lactate dehydrogenase A (2).] by binding to a hypoxia response element (HRE) (3).

Zhang et al. significantly increased our understanding of HIF-1α. Using mouse embryonic fibroblasts as a model, they found that knockout cell (KO) lines without HIF-1α consumed more oxygen and had higher ATP levels when compared to wild-type (WT) cells during both normoxia and hypoxia (1). Most interestingly, while WT cells exposed to hypoxia produced less reactive oxidative species (ROS) than normoxic WT cells, in KO cells exposure to hypoxia lead to an increase in ROS (1), suggesting that a major role of HIF-1α is in the attenuation of ROS production. Zhang et al. also demonstrated that hypoxia leads to mitochondrial autophagy and that this process is HIF-1 dependent and works through the BNIP3-Beclin-1-Atg5 pathway (1). Mitochondrial autophagy was thought to be a protective mechanism that leads to less ROS production. In addition, Hypoxia causes alteration of the cell calcium dynamics, which leads to alterations in many signaling pathways (12–14). A lack of Ca2+ signaling can trigger autophagy (15) and Ca2 + has been shown to regulate autophagy along with apoptosis (16–18).

While Zhang et al. have made major contributions to our understanding of adaptive responses to hypoxia (as evidenced by the 2019 Nobel prize in medicine being shared by William Kaelin, Sir Peter Ratcliffe, and Gregg Semenza for their work in this field), important questions remain. Does HIF-1α exert an influence on [Ca^2+^]_*i*_ levels? Is the role of HIF-1 in attenuating oxidative stress universal across all cell types? How do anti-oxidant defenses (exogenous or endogenous) impact HIF-1α expression? Is HIF-1α the only pathway that drives autophagy *via* the BNIP3-Beclin-1-Atg5 pathway? The purpose of this study was to answer these questions. Our primary hypothesis was that in the setting of hypoxia, HIF-1α inhibition would lead to increased intracellular calcium-mediated by reduced expression of L-type voltage-dependent Ca channels.

## Materials and Methods

### Cell Culture and Reagents

Rat (*Rattus norvegicus*) heart/myocardium cells (H9c2; #CRL-1446, ATCC, Manassas, VA, United States) were cultured and maintained following standard provider protocol. H9c2 cells (1 × 10^6^ cells/ml) were cultured in complete cell culture media [ATCC-formulated Dulbecco’s Modified Eagle’s Medium (DMEM, # 30-2002) supplemented with 10% fetal bovine serum (Sigma, United States)] and were incubated in a humidified atmosphere with 5% CO2 at 37°C, and allowed to grow up to 70–80% confluence before exposure to hypoxia or normoxia. When present, 200 μM (19–21) of Cobalt (II) chloride [(CoCl2), # C8661, Sigma, United States] a Hypoxia-inducible transcription factor 1 α (HIF-1 α) inducer, 1 mM of Dimethyloxalylglycine [(DMOG); Sigma, United States, # 400091] a HIF-Hydroxylase Inhibitor, 100 μM (22) of L-Ascorbic acid 2-phosphate sesquimagnesium salt hydrate [(A2P), #8960, Sigma, United States] an essential supplement and reactive oxygen species suppressant (23) and 1 *n*M (22, 24, 25) of Echinomycin (ECM, #SML0477, Sigma, United States) an antitumor antibiotic and potent hypoxia-inducible factor 1α (HIF-1α) the inhibitor was directly added to the culture medium.

### Cobalt Chloride and Hypoxia Mediated HIF-1α Induction

To mimic hypoxic conditions, H9c2 cells were cultured at a density of 1 × 10^6^ per ml on 100 × 30-mm Petri dishes/6 well plates as described above. In the CoCl_2_ experimental group, H9c2 cells were cultured in a complete culture medium supplemented with 200μM CoCl2 alone or with ECM, A2P for 6 h, at atmospheric conditions of 95% air and 5% CO2 at 37°C in a humidified incubator (21%O2). In the hypoxic experimental group, H9c2 cells were cultured under Hypoxia (1%O2) in a complete culture medium supplemented with ECM or A2P and were maintained in a modular incubator chamber flushed with a gas mixture containing 1%O2, 5% CO2, and 94% N2 at 37°C for 24 h. For controls, H9c2 cells were cultured in a complete culture medium under normoxia conditions. After incubation, cells were harvested immediately for analysis or preserved at –80°C freezer for future studies. Cell culture environment, reagent concentrations and specificity of all experimental groups are listed in Table 1.

**TABLE 1:** Experimental groups and cell culture environment.

Culture Environment	21% O2	Cocl2 (21% O2)			Hypoxia (1%O2)				21% O2
Experimental Groups	Control	Cocl2	Cocl2+A2P	Cocl2+ECM	1%O2	1%O2+ A2P	1%O2+ ECM	Si-Becn1	Si-Becn1
	1	2	3	4	5	6	7	8	9
Cocl2 (200μM)	–	+	+	+	–	–	–	–	–
A2P (100μM)	–	–	+	–	–	+	–	–	–
ECM(1nM)	–	–	–	+	–	–	+	–	–
Si-RNA (25 nM)	–	–	–	–	–	–	–	+	+
**Cell Culture and reagents**									
Culture Media	ATCC-formulated Dulbecco’s Modified Eagle’s Medium (DMEM, # 30-2002) supplemented with fetal bovine serum (Sigma, USA)								
Cocl2	Cobalt chloride (CoCl2), a commonly used hypoxia-mimetic agent, which induces hypoxia-like responses, (accumulation of HIF-1α protein), can block the degradation, and stabilizes hypoxia inducible factors (accumulation of HIF-1 protein).								
A2P	L-Ascorbic acid 2-phosphate sesquimagnesium salt hydrate (A2P),, is an oxidation resistant analog of Ascorbic acid, Studies suggest that A2P (25-250 μM) can effectively reduce the HIF-1α protein and transcriptional activity								
ECM	Echinomycin (ECM) is an antitumor antibiotic and potent hypoxia inducible factor 1α (HIF-1α) inhibitor.								
Si-RNA (Beclin-1)#6246 -CST	Beclin 1, a tumor suppressor protein, acts as an initiator of autophagy in mammals. SignalSilence^®^ Beclin-1 siRNA II (Cell Signaling Technology) specifically inhibit Beclin-1 expression.								

### siRNA Transfection

H9c2 cells (1 × 10^6^ cells/ml) were seeded in a 6-well plate containing a complete culture medium and cultured to 70–80% confluence before transfection. For knockdown of Beclin-1/Becn1 (Coiled-Coil, Moesin-Like BCL2-Interacting Protein), 10 μl of transfection reagent (TransIT-X2^®^ Dynamic Delivery System # MIR6000, Mirus) was diluted in 250 μl *OptiMEM* serum reduced medium, to this 25 nM of Beclin-1 siRNA was added and incubated at room temperature for 30 min to form transfection reagent: siRNA complex development. The transfection reagent: siRNA complex was then added to 70–80% confluence H9c2 cells (replaced with culture medium supplemented with 1%FBS) and incubated at 37°C with 5% CO2 in an incubator for 1 h. After 1 h incubation, culture medium FBS was increased to 10% (complete culture medium), and these pre- Beclin-1- siRNA transfected cells were used for mimicking hypoxic conditions. siRNA and transfection reagents concentrations were used according to the manufacturer’s instructions.

### Cell Viability Assay

Experimental groups (CoCl_2_ and Hypoxia) exposed H9c2 cells viability was measured using the CellTiter-Glo^®^ Luminescent cell viability assay kit (#G7570, Promega). Briefly, H9c2 cells were plated in 6-well plates and grown up to 70–80% confluence, then the plates were exposed to hypoxia or normoxia conditions, or Cobalt chloride as described in the methodology section. After incubation, H9c2 cells were incubated at room temperature for 15 min, and then, 100 μL of CellTiter-Glo reagent were added to cells and then placed on an orbital shaker for 2 min to induce cell lysis. Lysed cells were incubated at room temperature for 10 min to stabilize the luminescent signal and then using Synergy™ Multi-Mode Microplate Reader (BioTek, United States) luminescence was recorded. All the samples were read in duplicate. To represent the relative density of adhering cells (live cells) in culture dishes, we performed the crystal violet staining on CoCl2 and Hypoxia exposed H9c2 cells. In brief, after cells were exposed to hypoxic conditions, culture media was removed and the cells were washed with PBS (phosphate-buffered saline) and fixed for 20 min at RT with staining solution (0.05% w/v crystal violet, 1% formaldehyde, 1% methanol in 1x PBS). Fixed cells were rinsed with water and air-dried and images were obtained using a Nikon TS100 digital camera microscope.

### Measurement of Intracellular Calcium (Ca2+) Levels

Intracellular calcium (Ca2+) levels in CoCl2 and Hypoxia exposed H9c2 cells were assessed using the Fluo-4 NW calcium assay kit (# F36206, Molecular Probes, Invitrogen), by following the manufacturer’s protocol. Briefly, after cells were exposed to CoCl2 or hypoxia conditions, culture media was removed to avoid the source of baseline fluorescence then quickly 100-μl of Fluo-4 NW assay reagent was added and incubated for 30 min at 37°C, then incubated for an additional 30 min in the dark at room temperature. The relative fluorescence units (RFU) emitted by the Fluo-4-NW dye were quantified using a Synergy™ Multi-Mode Microplate Reader (BioTek, United States), excitation at 494 nm, and emission at 516 nm.

### Measurement of Cellular ATP Levels

Intracellular energy (ATP) levels were measured using the Bioluminescent Assay kit (MAK135, Sigma) according to the manufacturer’s protocol and as previously described (26). The addition of luciferase and D-Luciferin (ATP assay buffer) to the cells allowed for the measurement of the luminescent intensity of the sample (which was proportional to the amount of ATP) using Synergy™ Multi-Mode Microplate Reader, BioTek, United States. All the samples were read in duplicate and purified ATP (Abcam, ab 83355) was used as a standard.

### Measurement of Cellular NADP/NADPH Ratio

Changes in the NADP/NADPH ratio were evaluated using an NADP/NADPH -Glo assay kit (# G9081, Promega). Briefly after exposing cells to CoCl2 or hypoxia conditions, culture media was removed then quickly 50-μl of PBS and 50-μl of NADP/NADPH -Glo assay reagents were added, gently mixing the plate, and incubated for 30 min in dark at room temperature. After 30 min, NADP/NADPH ratios were (luminescence) measured using Synergy™ Multi-Mode Microplate Reader (BioTek, United States). All the samples were read in duplicate.

### Quantification of Intracellular Total Reactive Oxidative Species, Oxidative Stress (Protein Carbonylation, Malondialdehyde), and Antioxidant (Superoxide Dismutase, Catalase) Levels

The myocardium (H9C2 cells) total ROS, oxidative stress, and antioxidant levels toward hypoxia, HIF-1α inhibition, and under different treatments were measured using commercially available assay kits by following the manufacturer’s protocol. Intracellular total ROS levels were measured using a 2′-7′-dichlorofluorescein diacetate-based total ROS detection kit (#ENZ-51011, Enzo Life Sciences). Oxidative stress levels were assayed using Protein Carbonyl assay using the bioluminescent assay kit (MAK135; Sigma-Aldrich, United States) by following the manufacturer’s protocol and as previously described by *Osuru et al*. H9C2 cells Lipid peroxidation level was determined using the thiobarbituric acid (TBA) method with Lipid Peroxidation [malondialdehyde (MDA)] assay kit (MAK085; Sigma-Aldrich United States). H9C2 cell’s antioxidant levels were measured by quantifying the total SOD (Superoxide dismutase) levels using the SOD colorimetric activity kit (#EIASODC; Life technologies corporation-Invitrogen, United States) and by estimating the catalase (CAT) protein levels using western blotting method (for antibody concentration and methodology please see the western blotting section).

### Measurement of Cellular Hypoxia/Oxidative Stress Levels in Cells

Cellular hypoxia/oxidative stress levels were evaluated using a ROS-ID^^®^^ hypoxia/oxidative stress detection kit (#ENZ-51042, Enzo Life Sciences). H9c2 cells were cultured and maintained as described above in 8- chambered cell culture slides (Falcon #08-774-208, Fisher Scientifics) and allowed to grow up to 70–80% confluence, then the cells were exposed to CoCl2 or hypoxia conditions. After cells were exposed to CoCl2 or hypoxia, culture media was removed and cells were washed gently twice with PBS then ROS-ID^®^ hypoxia/oxidative stress detection solution was added and then cells were re- incubated under normal tissue culture conditions for 30 min. The detection solution was carefully removed, cells were washed twice with PBS, nuclei were stained with Hoechst 33342, then the slides were covered with a coverslip and a fluorescence microscope (Olympus, BX51) was used to capture the images using Green Filter for Oxidative stress and Texas Red Filter for Hypoxia detection. Data were normalized with nuclei stain (Hoechst 33342) fluorescence intensity and expressed as changes in fluorescence intensity.

### mRNA Analysis: Reverse Transcription-Quantitative Polymerase Chain Reaction

Total RNA was isolated from H9c2 cells in each experimental condition using a commercial total RNA isolation kit by following the manufacturer’s protocol (RNeasy plus mini kit, Qiagen) and the extracted RNA was quantified using a spectrophotometer- NanoDrop (Thermo Fisher Scientific, Inc). Total RNA (2–5 μg) was reverse transcribed to cDNA using the iScript-Adv cDNA Synthesis Kit according to the manufacturer’s recommendation (BioRad, United States). The cDNA (50–100 ng) was used for real-time PCR analysis in a final volume of 20 μl containing, iTaq universal SYBR^®^ Green supermix (BioRad, United States) and specific gene primers (Supplementary Table 1). qPCR was performed using the CFX Connect Real-time PCR system (BioRad, United States). Fold changes in expression were calculated using the 2- ΔΔCt method (27). Each reaction was run in duplicate or triplicate and Hprt1, or α-Tubulin was used as a normalization control.

### Analysis of Protein Expression: Western Blotting

The sources of antibodies were as follows: from Cell Signaling Technology Inc: Hypoxia-inducible factor 1-alpha (HIF1α, #4179,1:2000), Hypoxia-inducible factor 2-alpha (Hif2α, #7096,1:1500), Hypoxia-inducible factor 1-beta (HIF1β, #5537,1:2000). Autophagy Antibody Sampler Kit #4445 (Beclin-1,#3495,1:2000), Autophagy Related-5 (Atg5,#12994,1:2000), Autophagy Related-12 (Atg12,#4180,1:1000), Atg16L1,#8089,1:1000), heme-oxygenase-1 (HO-1,#86806,1:3000) and nitric oxide synthase (iNOS, #13120,1:1000). From Novus Biologicals: B-cell lymphoma 2 (bcl2 #NB100, 1:4000) and BCL2 Interacting Protein 3 Antibody (BNIP3,#NBP2-61715, 1:2000). From GeneTex Inc: BCL Interacting Protein 3L (BNIP3L,#GTX111876, 1:3000) Calcium Voltage-Gated Channel Subunit Alpha1 S (CACNA1S or Cav1.1,#GTX16634,1:500), Calcium Voltage-Gated Channel Subunit Alpha1 C (CACNA1C or Cav1.2,#GTX16608,1:500), Calcium Voltage-Gated Channel Subunit Alpha1 D (CACNA1D or Cav1.3,#GTX54755, 1:500), Superoxide Dismutase 1 antibody (SOD1,#GTX57658,1:3000) and Catalase antibody (CAT,#GTX110704, 1:2000). From Proteintech Group Inc: Mechanistic Target of Rapamycin Kinase (mTOR,#66888, 1:7000), AKT Serine/Threonine Kinase (AKT,#60203,1:6000), AMP-activated protein kinase (AMPK,#66536,1:3000), Vascular endothelial growth factor-a (VEGFA or VEGF,#66828,1:2000), Erythropoietin (EPO,#66975,1:3000) and loading control β-actin antibody (#66009,1:7000).

Selected protein expression levels were estimated using Western blot analysis by following previous reports with slight modification (26). H9c2 cells from each experimental condition were harvested, and their proteins were extracted either with RIPA Lysis Buffer containing Halt™ protease and phosphatase inhibitor (#89901,#78442 Thermo Fisher Scientific) or using nuclear protein extraction buffer (Abcam #ab113474), depending on the protein to be analyzed. We added 100 uM of the prolyl hydroxylase stabilizer CoCl2 or 1 mM of DMOG along with protease and phosphatase inhibitor to the extraction buffer to prevent hypoxia-inducible factor-1α (HIF-1 α) degradation in the presence of atmospheric oxygen. Total protein concentration was quantified using the Bicinchoninic Acid (BCA) kit (Thermo Scientific). 10–40 μg of whole-cell or nuclear proteins was heat-denatured at 95*^C^* and were separated using 4–20% Tris-Glycine polyacrylamide gradient gels (Bio-Rad). Proteins were transferred to PVDF membranes (Millipore) and residual protein sites were blocked with SuperBlock phosphate-buffered saline (PBS) Blocking Buffer (Thermo-Fisher #37515) and then incubated overnight with primary antibodies at the recommended concentrations at 4°C. Membranes were washed and incubated with horseradish peroxidase (HRP) conjugated secondary antibodies anti-mouse or anti-rabbit (1:15,000, depending on the primary antibody used) antibodies at room temperature. Membranes were washed then the antigen-antibody complexes were visualized using Super Signal West Femto enhanced chemiluminescence substrate (Thermo Scientific). Images were captured using GBOX (Chemi XR5; Syngene), and the intensity of the bands corresponding to the protein expression level was quantified with the computerized image analysis software (Gene Tools, Syngene). Target protein expression levels were normalized to the housekeeping protein levels (β-actin) and changes in protein expression levels were presented as ratios.

### Immunofluorescence

To illustrate the Cocl2 and hypoxia-induced intracellular Ca^2+^levels in H9c2 cells in addition to mRNA and protein analysis, we also used the immunofluorescence staining technique for Cav1.1 (1:200), Cav1.2 (1:200), and Cav1.3 (1:200) proteins, by following previous reports with slight modification (28). Briefly, H9c2 cells were cultured and maintained as described above in 8- chambered cell culture slides (Falcon #08-774-208, Fisher Scientific). After cells were exposed to CoCl2 or hypoxia, culture media was removed and cells were fixed with 4% formaldehyde at room temperature for 10 min and then incubated for 20 min with permeabilizing solution (PBS, 0.1% Triton X-100, and 1% FBS). The cells were washed with PBS and then blocked with 5% fetal bovine serum (FBS) and 1% BSA for 30 min and incubated with primary antibodies against Cav1.1, Cav1.2, and Cav1.3 (GeneTex Inc) overnight at 4°C. The following day cells were washed three times with PBS and then incubated with Alexa Fluor 488–conjugated donkey anti-rabbit secondary antibody (1:1000, Life Technologies) for 1 h at room temperature. Hoechst 33342 was used to stain the nuclei for 5 min, then the slides were covered with a coverslip and Fluoromount-G™ Mounting Medium. Representative images were acquired using a fluorescence microscope (Olympus, BX51), and Mean Fluorescence intensity (MFI) counts were calculated using Fiji: an open-source platform for biological-image analysis (29).

### Autophagy Analysis

In addition to ATG5, Becn1 mRNA, and protein analysis, we also used Cyto-ID Autophagy detection assay (#ENZ-KIT175, Enzo Life Sciences, United States) to detect autophagy in CoCl2 or hypoxia treated H9c2 cells. Briefly, H9c2 cells were cultured in 8-chambered cell culture slides and exposed to CoCl2 or hypoxia conditions. After cells were exposed to CoCl2 or hypoxia, cells were incubated with fluorescent Cyto-ID dye at 37°C for 30 min followed by nuclei stain-Hoechst 33342 for 5 min. After incubation, representative images were acquired using a fluorescence microscope (Olympus, BX51).

### Mitochondrial DNA Quantification

Mitochondrial DNA (mtDNA) levels of the H9C2 cells were quantified by following previously reported methods with slight modification (26). H9C2 cell genomic DNA from each experimental condition was extracted using DNeasy Blood & Tissue kit (#69504, Qiagen). To estimate the amount of mtDNA relative to nuclear DNA (nDNA), we used two sets of primers encoding a mitochondrial gene (*Mt_Nd1* and *Mt_Nd6*) and a nuclear gene Tubulin (*Tuba1*) (30). 100 ng of H9C2 cells DNA and iTaq universal SYBR^®^ Green supermix (BioRad) were used for real-time PCR analysis. Gene expression (Fold changes) was calculated using the 2-ΔΔCt method (27), each reaction was run in duplicate or triplicate, and α-Tubulin was used as a normalization control.

### Statistical Comparisons

All the data groups mRNA, protein expression, Mitochondrial DNA levels, ATP, ROS, NADP/NADPH ratios, and cell viability assays, control Vs experimental groups (Cocl2, Cocl2 + A2P, Cocl2 + ECM, 1%O2, 1%O2 + A2P, 1%O2 + ECM), and siRNA groups (si-Beclin1 + 21%O2, si-Beclin1 + 1% O2), were compared using the two-way ANOVA (or Mixed model) multiple comparisons compare groups column means (main column effect) with 0.05 (95% confidence interval). All calculations were performed using GraphPad Prism 8 (GraphPad Software, La Jolla, CA). Unless otherwise noted, each experiment was performed at least two to three times (for microplate-based assays, and qPCR assays each sample was run in duplicate or triplicate). All the data are shown in mean ± S.D, Symbols *, **+**, **#** and **≠** denotes significant, “ns” represents not significant by two-way ANOVA- multiple comparisons test. P-values are *^ns^p* > 0.05, *p < 0.05, **p < 0.01, ***p < 0.001 and ****p < 0.0001 compared to Control; ^+^*p* < 0.05, ^++^*p* < 0.01, ^+ + +^*p* < 0.001 and ^+ + + +^*p* < 0.0001 compared to Cocl2; ^#^*p* < 0.05, ^##^*p* < 0.01, ^###^*p* < 0.001 and ^####^*p* < 0.0001 compared to Hypoxia; ^≠^*p* < 0.05, ^≠^^≠^*p* < 0.01, ^≠^^≠^^≠^*p* < 0.001 and ^≠^^≠^^≠^^≠^*p* < 0.0001 compared to *si-becn1* (21%O2) Vs, *si-becn1* (1%O2).

## Results

### HIF-1α and Intracellular Ca^2+^

**FIGURE 1:**
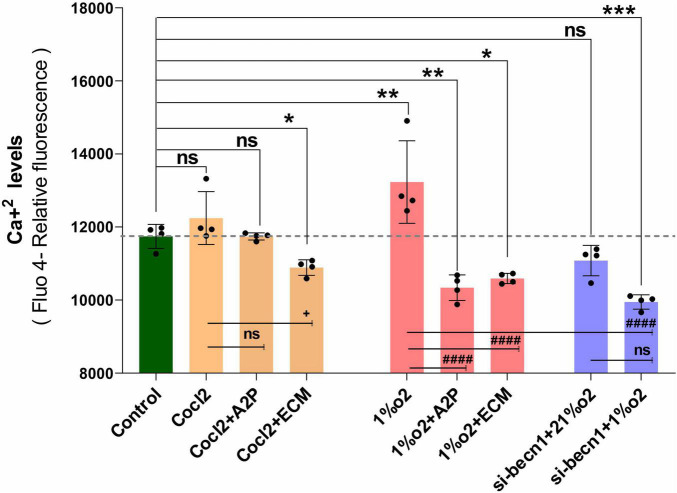
H9c2 cells grown under Hypoxia expressed decreased intracellular Calcium ([Ca^2+^]*i*) levels. The graph indicates the (Fluo-4 NW calcium assay) relative fluorescence changes of intracellular calcium levels in H9c2 cells treated with different molecules (ECM, A2P, siRNA-Beclin1) grown under CoCl2, Hypoxia (1%O2) and Normoxia conditions (21%O2). Hypoxia increased the cellular Ca2 + levels (**p < 0.003) compared to controls and was decreased in the cells treated with antioxidant-A2P (**p < 0.006) and in HIF-1 inhibitor- ECM group (*p < 0.028). Beclin-1 inhibition reduced the Ca2 + levels in hypoxic (1% O_2_) cells, ***p < 0.0005, but not in normoxia. Ca^2+^ levels also decreased in Cocl2 + ECM group (*p < 0.028) and not changed significantly in Control vs. Cocl2, Cocl2 + A2P group. Compared to Hypoxia cells (Cells grown under only hypoxia-1%O_2_/without Echniomycin (HIF inhibitor), A2P (anti-oxidant), and small interfering RNA to beclin-1-inhibition), cells grown with Hypoxia (1%O2) + A2P (^####^*p* < 0.0001), (1%O2) + ECM (^####^*p* < 0.0001), and in Beclin-1 inhibition (^####^*p* < 0.0001), cells grown under Hypoxia expressed significant decrease in Ca2 + levels. Data represents, *n* = 4 independent samples/group; Bars are mean ± S.D. Symbols *, **+**, **#**, and **≠** denotes significant, “ns” represents not significant by two-way ANOVA- multiple comparisons test. P-values are *^ns^p* > 0.05, *p < 0.05, **p < 0.01, ***p < 0.001, and ****p < 0.0001 compared to Control; ^+^*p* < 0.05, ^++^*p* < 0.01, ^+ + +^*p* < 0.001, and ^+ + + +^*p* < 0.0001 compared to Cocl2; ^#^*p* < 0.05, ^##^*p* < 0.01, ^###^*p* < 0.001, and ^####^*p* < 0.0001 compared to Hypoxia; ^≠^*p* < 0.05, ^≠^^≠^*p* < 0.01, ^≠^^≠^^≠^*p* < 0.001, and ^≠^^≠^^≠^^≠^*p* < 0.0001 compared to *si-becn1* (21%O2) Vs, *si-becn1* (1%O2).

**FIGURE 2:**
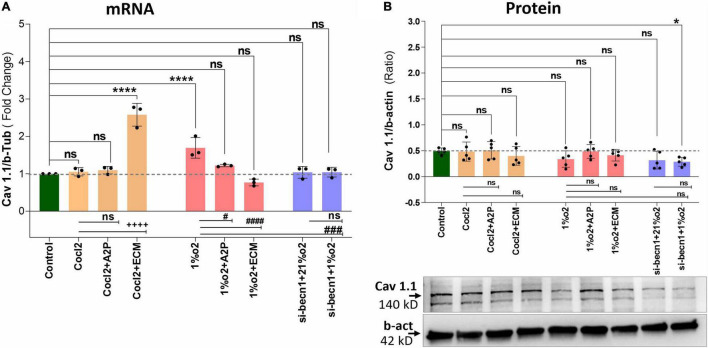
Hypoxia and Cocl2 exposure not significantly altered the H9c2 cells Cav1.1 (CACNA1S) mRNA and protein expression levels. **(A)** Cav1.1 mRNA expression levels, **(B)** Cav1.1 Protein expression levels, and representative western blots. Cav1.1 mRNA levels was increased in h9c2 cells exposed to Cocl2 + ECM (****p < 0.0001), and not changed significantly in Control vs. Cocl2, Cocl2 + A2P, 1%O2 + A2P, 1%O2 + ECM, and in Beclin-1 inhibition cells grown under normoxia and hypoxia conditions. Compared to Hypoxia (1%O2), cells exposed to 1%O2 + A2P (^#^*p* < 0.010), 1% O2 + ECM, (^####^*p* < 0.0001), and in Beclin-1 inhibition (^###^*p* < 0.0005) cells grown under Hypoxia expressed significant decrease in Cav1.1 mRNA levels. However, Cav 1.1 protein expression levels only decreased in (*p < 0.047) Beclin-1 inhibition cells under hypoxia and not significantly changed in all other experimental groups. Data represents, *n* = 3 (mRNA) and *n* = 5 (Protein) independent samples/group. Bars are mean ± S.D. Symbols *, **+**, **#**, and **≠** denotes significant, “ns” represents not significant by two-way ANOVA- multiple comparisons test. P-values are *^ns^p* > 0.05, *p < 0.05, **p < 0.01, ***p < 0.001, and ****p < 0.0001 compared to Control; ^+^*p* < 0.05, ^++^*p* < 0.01, ^+ + +^*p* < 0.001, and ^+ + + +^*p* < 0.0001 compared to Cocl2; ^#^*p* < 0.05, ^##^*p* < 0.01, ^###^*p* < 0.001, and ^####^*p* < 0.0001 compared to Hypoxia; ^≠^*p* < 0.05, ^≠^^≠^*p* < 0.01, ^≠^^≠^^≠^*p* < 0.001, and ^≠^^≠^^≠^^≠^*p* < 0.0001 compared to *si-becn1* (21%O2) Vs, *si-becn1* (1%O2). In western blots “→” represents a specific signal.

**FIGURE 3:**
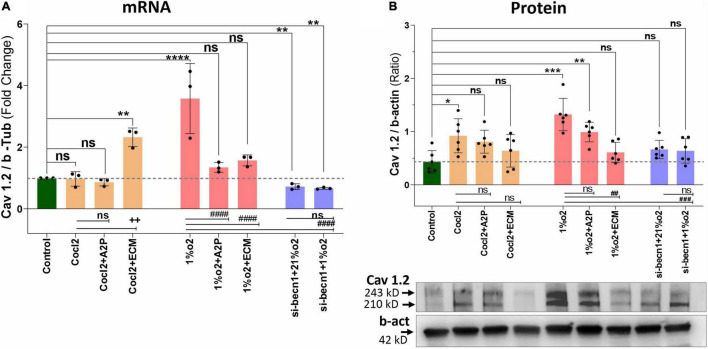
Hypoxia and Cocl2 exposure modifies the H9c2 cells Cav1.2 (CACNA1C) expression both at mRNA and protein level. **(A)** Cav1.2, mRNA expression levels, **(B)** Cav1.2 Protein expression levels and representative western blots. Compared to Controls Hypoxia increased the Cav1.2 expression at mRNA (****p < 0.0001) and Protein level (***p < 0.001). In addition, Cav1.2 mRNA levels are increased in Cocl2 + ECM (**p < 0.002) exposed cells. Compared to Hypoxia (1%O2), cells exposed to 1%O2 + A2P (^####^*p* < 0.0001), 1%O2 + ECM (^####^*p* < 0.0001) and in Beclin-1 inhibition cells grown under Hypoxia expressed significant decrease in Cav1.2 mRNA (^####^*p* < 0.0001) and Protein (^###^*p* < 0.001) expression levels. Data represents, *n* = 3 (mRNA) and *n* = 5 (Protein) independent samples/group. Bars are mean ± S.D. Symbols *, **+**, **#,** and **≠** denotes significant, “ns” represents not significant by two-way ANOVA- multiple comparisons test. P-values are *^ns^p* > 0.05, *p < 0.05, **p < 0.01, ***p < 0.001 and ****p < 0.0001 compared to Control; ^+^*p* < 0.05, ^++^*p* < 0.01, ^+ + +^*p* < 0.001 and ^+ + + +^*p* < 0.0001 compared to Cocl2; ^#^*p* < 0.05, ^##^*p* < 0.01, ^###^*p* < 0.001, and ^####^*p* < 0.0001 compared to Hypoxia; ^≠^*p* < 0.05, ^≠^^≠^*p* < 0.01, ^≠^^≠^^≠^*p* < 0.001, and ^≠^^≠^^≠^^≠^*p* < 0.0001 compared to *si-becn1* (21%O2) Vs, *si-becn1* (1%O2). In western blots “→” represents a specific signal.

**FIGURE 4:**
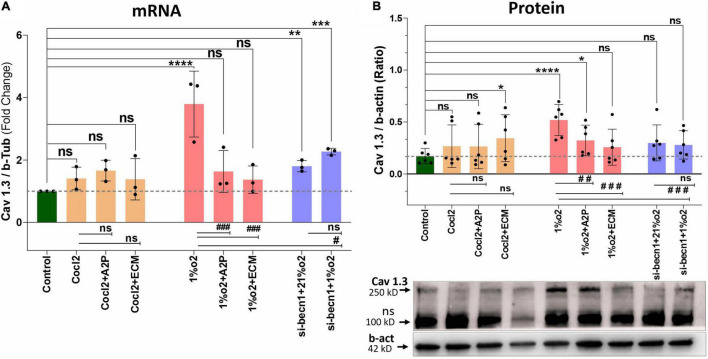
Hypoxia and Cocl2 exposure modifies the H9c2 cells Cav1.3 (CACNA1D) expression at mRNA and protein level. **(A)** mRNA expression levels of Cav 1.3, **(B)** Cav1.3 Protein expression levels and representative western blots. Hypoxia increased the Cav1.3 expression both at mRNA (****p < 0.0001) and Protein (****p < 0.008) level. In addition, Beclin-1 inhibition significantly increased the Cav1.3 mRNA levels both in normoxia (21%o2) **p < 0.001 and in hypoxia (1%O2) cells ***p < 0.0002, and protein expression levels was not changed significantly. Compared to Hypoxia cells Cav1.3 mRNA levels was decreased in 1%O2 + A2P (^###^*p* < 0.0005), 1%O2 + ECM (^###^*p* < 0.0001), and in Beclin-1 inhibition cells grown under Hypoxia (^#^*p* < 0.013) and protein expression levels also decreased in 1%O2 + A2P (^##^ *p* < 0.004), 1%O2 + ECM (^###^*p* < 0.001), and in Beclin-1 inhibition cells grown under Hypoxia (^###^*p* < 0.0003). Compared to control cells, Cocl2 exposure increased the Cav 1.3 protein expression levels in Cocl2 + ECM (*p < 0.022) group. No significant changes in Cav1.3 protein/mRNA expression level observed in all other groups compared to controls, Hypoxia and Cocl2. Data represents, *n* = 3 (mRNA) and *n* = 5 (Protein) independent samples/group. Bars are mean ± S.D. Symbols *, **+**, **#** and **≠** denotes significant, “ns” represents not significant by two-way ANOVA- multiple comparisons test. P-values are *^ns^p* > 0.05, *p < 0.05, **p < 0.01, ***p < 0.001 and ****p < 0.0001 compared to Control; ^+^*p* < 0.05, ^++^*p* < 0.01, ^+ + +^*p* < 0.001 and ^+ + + +^*p* < 0.0001 compared to Cocl2; ^#^*p* < 0.05, ^##^*p* < 0.01, ^###^*p* < 0.001 and ^####^*p* < 0.0001 compared to Hypoxia; ^≠^*p* < 0.05, ^≠^^≠^*p* < 0.01, ^≠^^≠^^≠^*p* < 0.001 and ^≠^^≠^^≠^^≠^*p* < 0.0001 compared to *si-becn1* (21%O2) Vs, *si-becn1* (1%O2). In western blots “→” represents a specific signal, “→ ns” represents a non-specific signal.

Exposure of H9c2 cells to hypoxia led to an increase in intracellular Ca^2+^ levels (Figure 1). Both A2P (antioxidant) and ECM (HIF-1 inhibitor) prevented this response. Interestingly, in cells exposed to siRNA inhibitor for beclin-1, exposure to hypoxia lead to a *reduction* in Ca^2+^ levels compare to controls. Exposure of H9c2 cells to hypoxia-mimetic agent cobalt chloride (CoCl_2_) and hypoxia (1%O_2_) did not change the Cav1.1 protein expression significantly (Figure 2). However, compare to controls Cocl2 + ECM and 1%O2 exposed h9c2 cells showed increased mRNA levels and decreased protein levels with Beclin1/becn1- inhibition cells grown under hypoxia (Figure 2). Exposure of H9c2 cells to hypoxia and Cocl2 exposure significantly modifies the Cav1.2 (CACNA1C) and Cav1.3 (CACNA1D) expression at mRNA and protein levels and these responses were blocked in presence of A2P and ECM (Figures 3, 4). Hypoxia increased the Cav1.2 expression at mRNA and protein level. Cav1.2 mRNA levels are increased in Cocl2 + ECM exposed cells and decreased in Beclin-1 inhibition cells grown under hypoxia and normoxia. In addition compared to hypoxia Beclin-1 inhibition cells grown under hypoxic conditions expressed significant decrease in Cav1.2 mRNA and the protein expression levels (Figure 3).

Hypoxia increased the Cav1.3 expression both at mRNA and protein level. In addition, Beclin-1 inhibition significantly increased the Cav1.3 mRNA levels both in normoxia and in hypoxia cells, and the protein expression levels were not changed. Hypoxia cells, Cav1.3 mRNA levels were decreased in 1%O2 + A2P, 1%O2 + ECM and in the Beclin-1 inhibition cells grown under Hypoxia. Protein levels also decreased in 1%O2 + A2P, 1%O2 + ECM, and in Beclin-1 inhibition cells grown under Hypoxia (Figure 4). These data suggest that in the setting of hypoxia, HIF-1 inhibition could lead to increased intracellular calcium and that, this is mediated by reduced expression of L-type voltage dependent Ca^++^ channels (Figures 1–4 and in Supplementary Figures 2A–F) in accordance with our primary hypothesis. In addition, the poor correlation between expression levels of mRNA and protein change possibly varied post-transcriptional mechanisms.

### HIF-1α and the Balance Between ATP and Reactive Oxidative Stress

**FIGURE 5:**
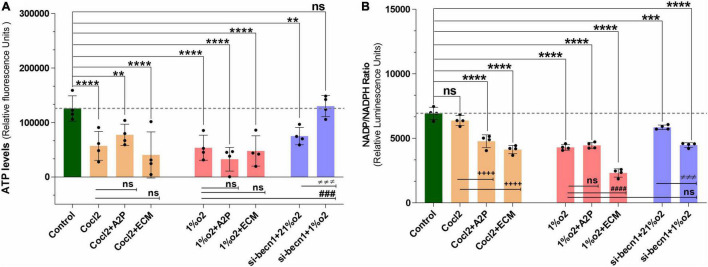
Hypoxia and Beclin1 inhibition decrease the H9c2 cells ATP levels and NADP/NADPH ratios. **(A)** The graph indicate the relative luminescence changes of ATP levels and **(B)** NADP/NADPH ratios in H9c2 cells exposed to CoCl2, Hypoxia (1%O2), Normoxia conditions (21%O2) and treated with ECM, A2P, siRNA-Beclin1. Compared to controls, ATP levels decreased in Cocl2 (****p < 0.0001), Cocl2 + A2P (***p < 0.009), Cocl2 + ECM (****p < 0.0001) and in hypoxia (1%o2) exposed cells (****p < 0.0001), 1%o2 + A2P (****p < 0.0001), 1%o2 + ECM (****p < 0.0001). In Beclin-1 inhibition cells, ATP levels are decreased under normoxia (21%o2) condition (***p < 0.0006), and no significant change was observed in Beclin-1-inhibition cells exposed to Hypoxia. However, compared to Hypoxia cells ATP levels, Beclin-1 inhibition cells grown under Hypoxia showed increased ATP levels (^###^*p* < 0.001). In H9c2 cells, compared to controls NADP/NADPH levels was decreased in Cocl2 + A2P (****p < 0.0001), Cocl2 + ECM (****p < 0.0001), hypoxia (1%O2) ****p < 0.0001, 1%O2 + A2P (****p < 0.0001) and in 1%O2 + ECM (****p < 0.0001) exposed cells. In beclin-1 inhibition cells, NADP/NADPH levels were decreased under normoxia (21%O2) condition (***p < 0.0008) and in Hypoxia exposure (****p < 0.0001). In addition, compared to Cocl2 exposed cells Cocl2 + A2P (^++++^*p* < 0.0001), Cocl2 + ECM (^++++^*p* < 0.0001) cells NADP/NADPH-ratio was further decreased and same way compared to Hypoxia, 1%O2 + ECM (^####^*p* < 0.0001) cells NADP/NADPH levels also decreased significantly. Data represents, n = 4 independent samples/group; Bars are mean ± S.D. Symbols *, +, #, and ≠ denotes significant, “ns” represents not significant by two-way ANOVA- multiple comparisons test. P-values are *^ns^p* > 0.05, *p < 0.05, **p < 0.01, ***p < 0.001 and ****p < 0.0001 compared to Control; ^+^*p* < 0.05, ^++^*p* < 0.01, ^+++^*p* < 0.001 and ^++++^*p* < 0.0001 compared to Cocl2; ^#^*p* < 0.05, ^##^*p* < 0.01, ^###^*p* < 0.001 and ^####^*p* < 0.0001 compared to Hypoxia; ^≠^*p* < 0.05, ^≠^^≠^*p* < 0.01, ^≠^^≠^^≠^*p* < 0.001 and ^≠^^≠^^≠^^≠^*p* < 0.0001 compared to si-becn1 (21%O2) Vs, si-becn1 (1%O2).

**FIGURE 6:**
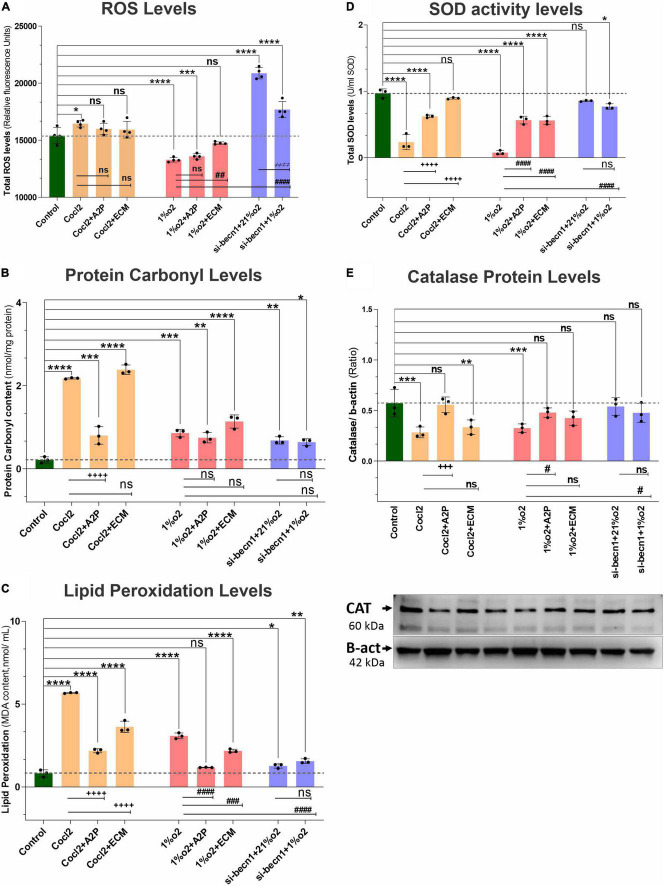
Hypoxia, Cocl2 exposure, and Beclin1 inhibition markedly alter the H9c2 cells oxidative stress and antioxidant levels. The graphs indicate oxidative stress markers **(A)** Total ROS levels, **(B)** Protein Carbonyl levels, **(C)** Lipid Peroxidation levels, **(D)** antioxidant marker SOD activity levels, and **(E)** Catalase Protein levels in H9c2 cells treated with ECM, A2P, siRNA-Beclin1 and grown under CoCl2, Hypoxia (1%O2) and Normoxia conditions (21%O2). Total- ROS levels decreased in hypoxia (1%O2) exposed cells (****p < 0.000), 1%O2 + A2P (****p < 0.0001), and not significantly changed in 1%O2 + ECM treated cells (^*ns*^*p* < 0.267). Cocl2 exposure (*p < 0.022) and Beclin-1 inhibition increased the total- ROS levels both in normoxia (21%O2), ****p < 0.0001, and in hypoxia (1%O2) ****p < 0.0001 cells, and not changed in Cocl2 + A2P, Cocl2 + ECM exposed cells. However, compared to Hypoxia cells ROS levels, 1%O2 + ECM cells ROS levels was increased significantly (^##^*p* < 0.001) and not changed in all other experimental groups. Protein carbonyl content, and Lipid peroxidation levels were increased both in Hypoxia and in Cocl2 exposed H9C2 cells compared to controls and they were decreased in antioxidant (Cocl2 + A2P) treated Cells. Anti-oxidant marker SOD and Catalase (Protein) levels were also significantly decreased in both CoCl2 and Hypoxia (1%O2) exposed myocardium cells, and this decrease was inhibited in A2P treated myocardium cells. Data represents, *n* = 3–4 independent samples/group; Bars are mean ± S.D. Symbols *, **+**, **#**, and **≠** denotes significant, “ns” represents not significant by two-way ANOVA- multiple comparisons test. P-values are *^ns^p* > 0.05, *p < 0.05, **p < 0.01, ***p < 0.001 and ****p < 0.0001 compared to Control; ^+^*p* < 0.05, ^++^*p* < 0.01, ^+ + +^*p* < 0.001 and ^+ + + +^*p* < 0.0001 compared to Cocl2; ^#^*p* < 0.05, ^##^*p* < 0.01, ^###^*p* < 0.001 and ^####^*p* < 0.0001 compared to Hypoxia; ^≠^*p* < 0.05, ^≠^^≠^*p* < 0.01, ^≠^^≠^^≠^*p* < 0.001 and ^≠^^≠^^≠^^≠^*p* < 0.0001 compared to *si-becn1* (21%O2) Vs, *si-becn1* (1%O2).

Exposure of H9c2 cells to hypoxia led to a decrease in intracellular ATP levels (Figure 5A), decreased NADP/NADPH levels (Figure 5B) and a sharp reduction in total ROS levels (Figure 6A). Neither A2P nor ECM affected the change in ATP with hypoxia. Though the impact of hypoxia on total ROS was reversed with HIF-1 inhibition through ECM (1%O2 vs. 1% O2 + ECM) interestingly, in normoxia, cobalt chloride (prolyl hydroxylase inhibitor) led to reduced ATP levels, similar to those seen during hypoxia and an increase in ROS (Control vs. Cocl2). In cells exposed to beclin-1 inhibitor, ATP levels increase during hypoxia compared to hypoxia cells (no beclin-1 inhibitor). Most notably, beclin-1 inhibition led to a profound increase in total ROS levels during normoxia, which was only partially attenuated during hypoxia (Figure 6A). In addition to the increased total ROS levels in Cocl2 and Beclin1 inhibition cells, we observed oxidative stress markers; total protein carbonyl content (Figure 6B) and Lipid peroxidation (Figure 6C) levels were significantly increased. The anti-oxidant marker SOD and Catalase levels were also considerably decreased in Hypoxia and in Cocl2 exposed H9C2 cells (Figures 6D,E). We observed that both A2P (anti-oxidant, oxidation-resistant derivative of ascorbic acid) and ECM (HIF-1α inhibitor) attenuated the hypoxia (1%O2) and hypoxia-mimetic agent (Cocl2) disturbed cells Oxidative and anti-oxidant responses to varying degrees (Figure 6).

In addition, we observed that Hypoxia leads to decreased NADP/NADPH levels (Figure 5B) which were not affected by A2P but appeared to be amplified with ECM. Exposure of cells to both CoCl_2_ and hypoxia leads to an increase in nitroreductase activity (hypoxia detector) as well as reactive species (Supplementary Figure 3). During exposure to CoCl_2_, the addition of the antioxidant A2P and HIF-1α inhibitor ECM leads to a reduction in nitroreductase activity as well as ROS. In cells exposed to hypoxia, A2P and ECM also lead to a reduction in nitroreductase activity but no influence on ROS levels.

### Impact of Anti-oxidants and HIF-1α Inhibitors on HIF-1α Expression

**FIGURE 7:**
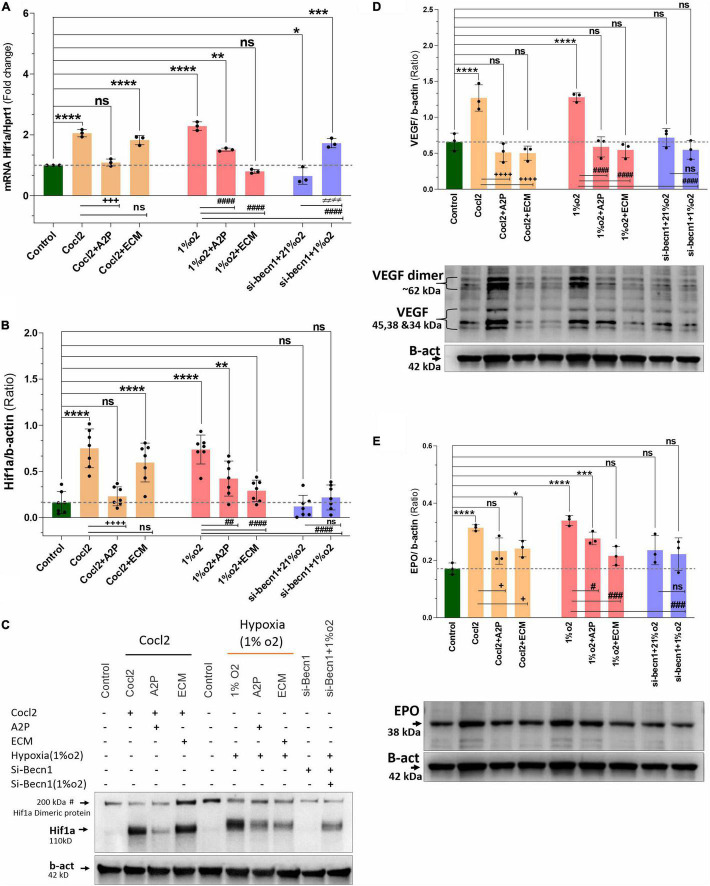
Hypoxia and Cocl2 exposure increase the H9c2 cells HIF1α and downstream targets VEGF and EPO expression levels. **(A)** Hif1α mRNA levels **(B)** Hif1α Protein expression levels **(C)** representative western blots showing Hif1α protein levels. **(D)** VEGF protein expression levels and representative western blots. **(E)** EPO protein expression levels and representative western blots. Compared to control cells, Hif1α mRNA increased in cells exposed to Cocl2 (****p < 0.0001), Cocl2 + ECM (****p < 0.0001), Hypoxia (1%O2) **p < 0.0001, and Hypoxia (1%O2) + A2P, **p < 0.001. Beclin-1 inhibition reduced the Hif1α mRNA levels in normoxia (21%O2) *p < 0.027 and increased in hypoxia (1%O2) exposed cells, ****p < 0.0001. In addition, compared to Cocl2 exposed cells, CoCl_2_ + A2P (^+ + +^*p* < 0.0004), compared to Hypoxia (1%O2) cells, 1%O2 + A2P (^####^*p* < 0.0001), 1%O2 + ECM (^####^*p* < 0.0001), and beclin-1 inhibition cells grown under Hypoxia (^####^*p* < 0.0001) expressed significantly decreased the Hif1α mRNA levels. As expected, Hif1α protein expression levels was increased in Cocl2 (****p < 0.0001), and in Hypoxia (****p = 0.0001) cells. Compared to controls, Cocl2 + ECM cells showed increased Hif1α protein expression levels (****p < 0.0001). Compared to Cocl2 exposed cells, Cocl2 + A2P cells Hif1α protein expression levels was decreased (^+ + + +^*p* < 0.0001). Also, compared to Hypoxia cells, 1%O2 + A2P (^##^*p* < 0.0012), 1%O2 + ECM (^####^*p* < 0.0001), and Beclin-1 inhibition cells grown under Hypoxia (^####^*p* < 0.0001) expressed decreased Hif1α Protein levels. HIF1α downstream target VEGF and EPO protein expression levels were significantly increased in Hypoxia and in Cocl2 exposed cells, and showed reduced expression levels with A2P (anti-oxidant) and ECM (Hif1 inhibition) treatment. Beclin-1 inhibition cells grown under normoxia and in hypoxia also showed the decreased VEGF and EPO protein expression levels compared to Hypoxia and Cocl2 exposed cells. Data represents, *n* = 3 (mRNA), *n* = 7 (Hif1a protein) and *n* = 3 (VEGF and EPO protein) independent samples/group; Bars are mean ± S.D. Symbols *, **+**, **#** and **≠** denotes significant, “ns” represents not significant by two-way ANOVA- multiple comparisons test. P-values are *^ns^p* > 0.05, *p < 0.05, **p < 0.01, ***p < 0.001 and ****p < 0.0001 compared to Control; ^+^*p* < 0.05, ^++^*p* < 0.01, ^+ + +^*p* < 0.001 and ^+ + + +^*p* < 0.0001 compared to Cocl2; ^#^*p* < 0.05, ^##^*p* < 0.01, ^###^*p* < 0.001 and ^####^*p* < 0.0001 compared to Hypoxia; ^≠^*p* < 0.05, ^≠^^≠^*p* < 0.01, ^≠^^≠^^≠^*p* < 0.001 and ^≠^^≠^^≠^^≠^*p* < 0.0001 compared to *si-becn1* (21%O2) Vs, *si-becn1* (1%O2). In western blots “→” represents a specific signal.

Exposure of H9c2 cells to hypoxia led to an increase in HIF-1α mRNA (Figure 7A) and protein expression (Figure 7B). Compared to hypoxia, in cells exposed to hypoxia + A2P lead to a reduction in HIF-1α expression both at mRNA, and at protein. In hypoxia (compared to no HIF-1 inhibition cells) HIF-1 inhibition through ECM significantly downregulates the H9c2 cell’s Hif-1α expression both at mRNA, and protein levels (Figures 7B,C). These data suggest that both A2P (anti-oxidant), and ECM (HIF inhibitor), inhibit the hypoxia-induced HIF-1α expression in myocardial (H9c2) cells. In addition compared to hypoxia, beclin-1 inhibition cells grown under hypoxic conditions also expressed significant decrease in HIF-1α mRNA and the protein expression levels (Figures 7A–C).

In addition to increased HIF1α expression, we observed that the HIF1α downstream targets VEGF (Vascular endothelial growth factor) and EPO (Erythropoietin) protein expression levels were also significantly increased in Hypoxia and in Cocl2 exposed cells and these overexpressed protein levels were ameliorated with A2P (anti-oxidant) and ECM (Hif1 inhibition) treatment. Beclin-1 inhibition cells grown under normoxia and in hypoxia conditions also expressed the decreased VEGF and EPO protein expression levels compared to Hypoxia and Cocl2 exposed cells (Figures 7D,E).

### HIF-1α and the Beclin-1 Pathway

**FIGURE 8:**
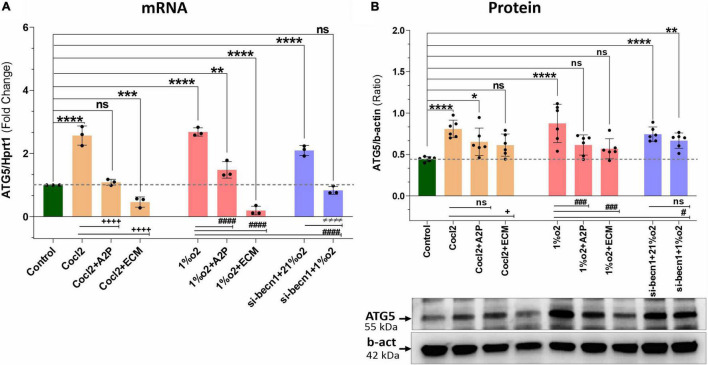
Hypoxia, Cocl2 exposure and Beclin1 inhibition increase the Autophagy protein ATG5 expression levels. **(A)** ATG5 mRNA expression levels, **(B)** ATG5 Protein expression levels and representative western blots. ATG5 mRNA levels was increased in h9c2 cells exposed to Cocl2 ****p < 0.0001, and decreased in Cocl2 + ECM (***p < 0.0004) treated cells. ATG5 mRNA levels increased in Hypoxia (1%O2) cells ****p < 0.0001, 1%O2 + A2P (**p < 0.001) exposed cells, but decreased in 1% O2 + ECM (****p < 0.0001) exposed cells. Beclin-1 inhibition increased the ATG5 mRNA levels in normoxia (21%O2) ****p < 0.0001 and unchanged in hypoxia (1%O2) exposed cells. In addition compared to Cocl2 cells, Cocl2 + A2P (^+ + + +^*p* < 0.0001), Cocl2 + ECM (^+ + + +^*p* < 0.0001) cells ATG5 mRNA levels also decreased. Compared to Hypoxia cells, 1%O2 + A2P (^####^*p* < 0.0001), 1%O2 + ECM (^####^*p* < 0.0001) and in beclin-1 inhibition cells grown under Hypoxia (^####^*p* < 0.0001) expressed significantly decreased ATG5 mRNA levels. Compared to control cells, ATG5 protein expression levels were increased in Cocl2 (****p < 0.0001), Hypoxia (****p < 0.0001) exposed cells. Beclin-1 inhibition cells exposed to normoxia (21%O2) ****p < 0.0001 and Hypoxia (**p < 0.005) showed increased ATG5 protein. In addition compared to Hypoxia cells, 1%O2 + ECM (^###^*p* < 0.001), 1%O2 + A2P (^###^*p* < 0.0006) and Beclin-1 inhibition cells (^#^ *p* < 0.02) express decreased ATG5 protein and no significant changes in ATG5 protein expression level. Data represents, *n* = 3 (mRNA) and *n* = 6 (Protein) independent samples/group. Bars are mean ± S.D. Symbols *, **+**, **#** and **≠** denotes significant, “ns” represents not significant by two-way ANOVA- multiple comparisons test. P-values are *^ns^p* > 0.05, *p < 0.05, **p < 0.01, ***p < 0.001 and ****p < 0.0001 compared to Control; ^+^*p* < 0.05, ^++^*p* < 0.01, ^+ + +^*p* < 0.001 and ^+ + + +^*p* < 0.0001 compared to Cocl2; ^#^*p* < 0.05, ^##^*p* < 0.01, ^###^*p* < 0.001 and ^####^*p* < 0.0001 compared to Hypoxia; ^≠^*p* < 0.05, ^≠^^≠^*p* < 0.01, ^≠^^≠^^≠^*p* < 0.001 and ^≠^^≠^^≠^^≠^*p* < 0.0001 compared to *si-becn1* (21%O2) Vs, *si-becn1* (1%O2). In western blots “→” represents a specific signal.

Exposure of H9c2 cells to hypoxia led to an increase in both ATG5 mRNA (Figure 8A) and protein expression (Figure 8B). Both A2P and ECM attenuated this response to varying degrees at mRNA and no change was observed in protein expression of hypoxia (1%O2 + A2P) group, this could be due to post-transcriptional mechanisms. In cells exposed to siRNA inhibitor for beclin-1, ATG5 mRNA and protein levels were increased compared to controls in normoxia (21%O2). Compared to hypoxia cells ATG5 mRNA, Beclin-1 inhibition cells grown under hypoxia expressed significantly less ATG5 mRNA and protein levels (Figures 8A,B). In addition to ATG5 levels, the Cyto-ID Autophagy detection marker staining shown in Supplementary Figure 2G, visually (in cells) demonstrates the impact of CoCl2, hypoxia, A2P, and ECM on autophagy (Mean Fluorescence intensity levels were shown in Supplementary Figure 2H).

**FIGURE 9:**
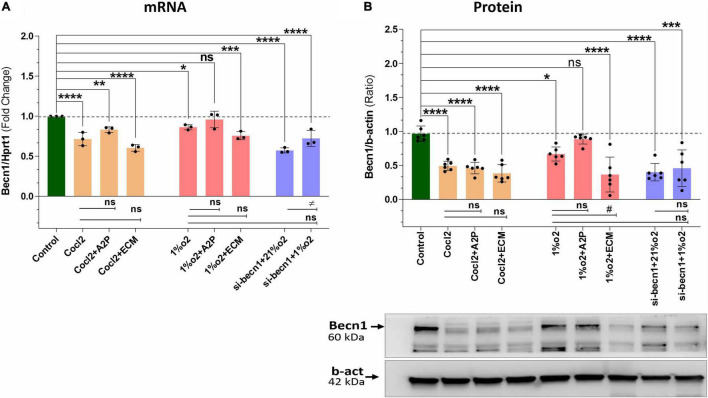
Hypoxia, Cocl2 exposure decrease the Autophagy protein-*Becn1* (*Beclin-1) expression* at mRNA and Protein level. **(A)** *Becn1* mRNA expression levels **(B),** *Becn1* protein expression levels and representative western blots showing *Becn1* protein levels in H9c2 cells treated with ECM, A2P, siRNA-Beclin1 and grown under CoCl2, Hypoxia (1%O2), and Normoxia conditions (21%O2). *Becn1* mRNA levels was decreased in h9c2 cells exposed to Cocl2 (****p < 0.0001), Cocl2 + ATP (**p < 0.004) and Cocl2 + ECM (****p < 0.0001). *Becn1* mRNA levels decreased in Hypoxia cells (1%O2) (*p < 0.019) and in Hypoxia (1%O2) + ECM, ***p < 0.0001. Beclin-1 inhibition decreased the *becn1* mRNA levels in normoxia (21%O2) ****p < 0.0001 and in hypoxia (1%O2) (****p < 0.0001) exposed cells. *Becn1* protein expression levels also decreased in Cocl2 (****p < 0.0001), Cocl2 + A2P (****p < 0.0001), Cocl2 + ECM (****p < 0.0001), and in Hypoxia (1%O2) *p < 0.026, and in 1%O2 + ECM (****p < 0.0001). Beclin-1 inhibition confirms the decreased *Becn1* protein under normoxia (21%O2) exposure (****p < 0.0001) and in Hypoxia (***p < 0.0003). Data represents, *n* = 3 (mRNA) and *n* = 6 (Protein) independent samples/group; Bars are mean ± S.D. Symbols *, **+**, **#** and **≠** denotes significant, “ns” represents not significant by two-way ANOVA- multiple comparisons test. P-values are *^ns^p* > 0.05, *p < 0.05, **p < 0.01, ***p < 0.001, and ****p < 0.0001 compared to Control; ^+^*p* < 0.05, ^++^*p* < 0.01, ^+ + +^*p* < 0.001, and ^+ + + +^*p* < 0.0001 compared to Cocl2; ^#^*p* < 0.05, ^##^*p* < 0.01, ^###^*p* < 0.001, and ^####^*p* < 0.0001 compared to Hypoxia; ^≠^*p* < 0.05, ^≠^^≠^*p* < 0.01, ^≠^^≠^^≠^*p* < 0.001, and ^≠^^≠^^≠^^≠^*p* < 0.0001 compared to *si-becn1* (21%O2) Vs, *si-becn1* (1%O2). In western blots “→” represents a specific signal.

**FIGURE 10:**
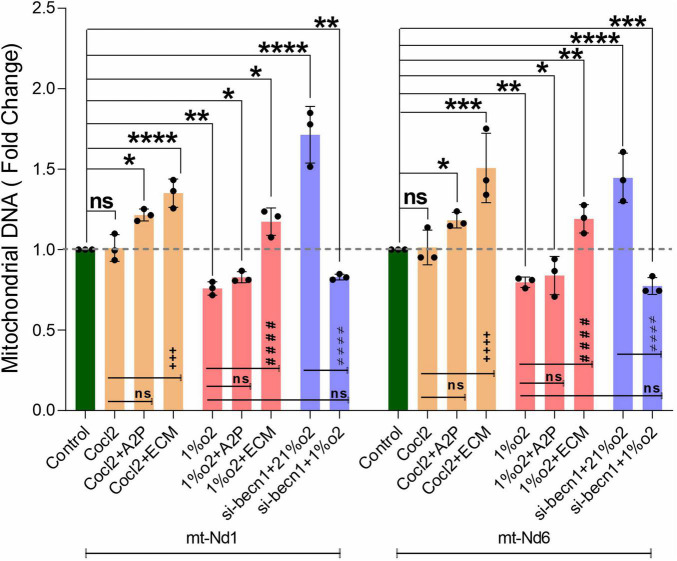
Hypoxia decrease the H9c2 cells Mitochondrial DNA levels. The graph indicates the Mitochondrial DNA encoded genes (*Mt-Nd1* and *Mt-Nd6*) levels in H9c2 cells treated with ECM, A2P, siRNA-Beclin1 and grown under CoCl2, Hypoxia (1%O2), and Normoxia conditions (21%O2). Mitochondrial DNA (*Mt-Nd1* and *Mt-Nd6*) levels were increased significantly in Cocl2 + A2P, Cocl2 + ECM, and in Hypoxia + ECM groups, and the Mt-DNA levels were decreased in Hypoxia (1%O2), Hypoxia (1%O2) + A2P groups. Beclin-1 inhibition cells Mt-DNA (*Mt-Nd1* and *Mt-Nd6*) levels increased in normoxia (21%O2) and were decreased in hypoxia (1%O2) exposed cells. Compared to Hypoxia cells, 1%O2 + ECM cells express increased Mt-DNA levels. Mitochondrial DNA levels was normalized to nuclear DNA levels (Tubulin A1) and relative Mt-DNA levels are expressed as fold change to controls. Data represents *n* = 3 independent samples/group; Bars are mean ± S.D. Symbols *, **+**, **#** and **≠** denotes significant, “ns” represents not significant by two-way ANOVA- multiple comparisons test. P-values are *^ns^p* > 0.05, *p < 0.05, **p < 0.01, ***p < 0.001 and ****p < 0.0001 compared to Control; ^+^*p* < 0.05, ^++^*p* < 0.01, ^+ + +^*p* < 0.001 and ^+ + + +^*p* < 0.0001 compared to Cocl2; ^#^*p* < 0.05, ^##^*p* < 0.01, ^###^*p* < 0.001 and ^####^*p* < 0.0001 compared to Hypoxia; ^≠^*p* < 0.05, ^≠^^≠^*p* < 0.01, ^≠^^≠^^≠^*p* < 0.001 and ^≠^^≠^^≠^^≠^*p* < 0.0001 compared to *si-becn1* (21%O2) Vs, *si-becn1* (1%O2).

We observed a slight reduction in *Becn1* or beclin-1 mRNA and protein expression with exposure of H9c2 cells to hypoxia (Figure 9). *Becn1* mRNA levels decreased in hypoxia cells and in hypoxia + 1%O2 + ECM cells compared to control. Beclin-1 inhibition decreased the *Becn1* mRNA levels in normoxia, and in hypoxia (1%O2) exposed cells. In addition, *Becn1* protein expression levels also decreased in Hypoxia and in Hypoxia (1%O2) + ECM groups. Beclin-1 inhibition also confirms the decreased *Becn1* protein under normoxia (21%O2) exposure and in Hypoxia (Figure 9). Exposure of H9c2 cells to hypoxia led to a decrease in mitochondrial DNA (Mt-nd1, Mt-nd6) levels (Figure 10), whereas application of beclin-1 siRNA significantly increased the mitochondrial DNA, which somewhat attenuated when exposed to hypoxia. In addition to mainly studied protein targets (Hif1α, ATG5, and Beclin1), several hypoxia, oxidative stress, and autophagy responsive proteins (ATG12, ATG16L, SOD1, HO-1, iNOS, BCL2, Bnip3, Bnip3l, Hif2a, Hif2b, mTOR, AKT Protein AMPK) expression levels in Myocardium (H9C2 cells) is shown in Supplementary Figure 4.

## Discussion

**FIGURE 11:**
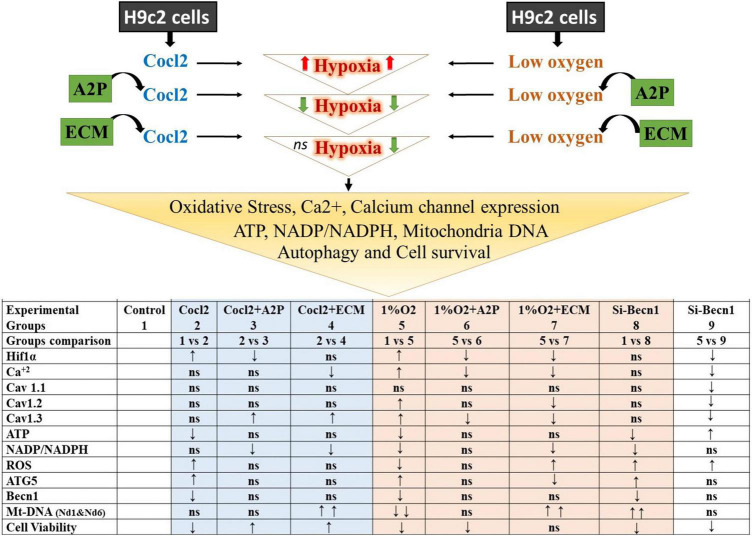
Summary of the major observed Molecular and Cellular Response of Heart; Myocardium (H9c2) cells toward chemical (CoCl2)-and low oxygen Induced Hypoxia. The results shown for Hif1α, Cav 1.1, Cav 1.2, Cav1.3, ATG5, and Becn1 are protein expression levels compared to control and experimental groups. ↑ increased; ↓ decreased; *ns*, not significant/no change. Hypoxia-inducible factor 1-alpha (Hif1α); Calcium (Ca2 +); calcium channel, voltage-dependent, L-type, alpha- 1S subunit, (CACNA1S) or (Cav 1.1); Calcium channel, voltage-dependent, L type, alpha-1C subunit (CACNA1C) or (Cav1.2); Calcium channel, voltage-dependent, L-type, alpha-1D subunit, (CACNA1D) or (Cav1.3); Adenosine triphosphate (ATP); Nicotinamide adenine dinucleotide phosphate (NADP)/Reduced nicotinamide adenine dinucleotide phosphate (NADPH); Reactive oxygen species (ROS); ATG5 (Autophagy Related 5); Beclin-1, Autophagy Related (Becn1); Mt-Nd1, mitochondrially encoded NADH dehydrogenase-1; Mt-Nd6, mitochondrially encoded NADH dehydrogenase 6.

H9c2 cells grown under hypoxia expressed decreased intracellular Calcium ([Ca^2+^]*i*) levels (Figure 1). Hypoxia and Cocl2 exposure did not alter the Cav1.1 (CACNA1S) mRNA and protein expression levels in H9c2 cells but significantly altered the Cav1.2 (CACNA1C) and Cav1.3 (CACNA1D) expression both at mRNA and protein level (Figures 2–4). Hypoxia, Cocl2 exposure, and Beclin1 inhibition decreased the ATP levels in H9c2 cells (Figure 5A), and increased the oxidative stress (ROS) levels under normoxia and noticeably alter the H9c2 cells oxidative and antioxidant levels (Figure 6). NADP/NADPH ratio decreased in hypoxia, and Beclin-1 silenced H9c2 cells (Figure 5B). HIF1α and downstream targets VEGF and EPO protein expression levels (Figures 7D,E) and Hypoxia/Oxidative stress ratio levels (Supplementary Figure 3) are decreased in cells cultured with A2P (antioxidant). Hypoxia, Cocl2 exposure, and Beclin1 inhibition increased the autophagy protein ATG5 expression levels (Figure 8) and decreased the Becn1 expression (Figure 9) and mitochondrial DNA levels of H9c2 cells (Figure 10). The major molecular and cellular changes in Myocardium (H9c2) cells in response to chemical (CoCl2) and low oxygen-induced hypoxia were summarized in Figure 11.

### HIF-1α and Intracellular Ca^2+^

Li et al. demonstrated that cultured PC12 cells subjected to hypoxia increased [Ca^2+^]_*i*_ which is not unexpected based on previously published data showing increased [Ca^2+^]_*i*_ in both endothelial (31, 32) and PC12 (33) cells exposed to hypoxia as well as the intact rat brain (34). Li et al. also found that HIF-1α inhibition led to a decrease in the Cav1.2 gene (which encodes for an L-type voltage-dependent Ca^2+^ channel) which is normally known to be increased during hypoxia (32). They did not report the [Ca^2+^]_*i*_ levels in the HIF-inhibited cells (24). Presumably in the setting of low oxygen, increased [Ca^2+^]_*i*_ is maladaptive, and understanding whether or not HIF-1α plays a role in Ca^2+^ regulation is important.

Similar to what other authors have reported, we found that hypoxia exposure (1%O_2_) leads to a significant increase in [Ca^2+^]_*i*_ (Figure 1) and decreased cell viability (p < 0.019, Supplementary Figures 1A,B). Like Li et al., we found that inhibition of HIF-1α with ECM, attenuated Cav 1.2 (and Cav 1.3) expression, but we also observed a *reduction* in [Ca^2+^]_*i*_ with hypoxia + ECM. Given the clear adverse impact of excessive [Ca^2+^]_*i*_ on cell survival and the general pro-survival response of HIF-1α, this result is inquisitive. While not statistically significant, there was also a trend toward increased [Ca^2+^]_*i*_ in normoxic cells exposed to the PHD inhibitor, CoCl_2_. Additionally, normoxic cells exposed to the HIF-1α inhibitor ECM also produced a significant decrease in [Ca^2+^]_*i*_. Taken together, these results suggest that HIF-1α leads to *increased* [Ca^2+^]_*i*_, although there must be other mechanisms involved as the CoCl_2_ group exhibited a similar increase in HIF-1α as the hypoxic group but did not yield an appreciable increase in [Ca^2+^]_*i*_.

Interestingly, while uncontrolled [Ca^2+^]_*i*_ accumulation is thought to represent a “final common pathway” for hypoxic cell death (35), there is some evidence that *moderate* increases in [Ca^2+^]_*i*_ are protective, at least in hypoxia-tolerant organisms (36). Furthermore, increased [Ca^2+^]_*i*_ may play an essential role in both hypoxic and anesthetic preconditioning (37). Though L-type Ca2+ channels are more sensitive to hypoxia, changes in cellular Ca2 + levels will also be associated with the levels of other types of Ca2+ channels, such as the T-type Ca2+ channel (Cav3.1-3) (38). Sarcoplasmic/endoplasmic reticulum calcium ATPases (SERCA), Phospholamban (PLN) (39, 40), sodium-calcium exchange, plasma membrane Ca2 + ATPase (41, 42), will likely affect the Ca2 + levels along with extrinsic and intrinsic stimuli (43). More work is needed to better understand the role of HIF-1α in modulating the intracellular concentration of this important cation, especially in intact animals.

### HIF-1α and the Balance Between ATP and Reactive Oxidative Stress

The relationship between O_2_ availability, ROS/RNS, oxidative stress, and high energy phosphates (ATP) is unclear. Zhang et al. exposed mouse embryonic fibroblast (MEFs) to hypoxia (1%O_2_) and found that hypoxic MEFs deficient in HIF-1α exhibited *higher* levels of O_2_ consumption, ATP, and ROS than wild-type (WT) cells exposed to 20% O_2_ (1). It is worthy of notice how the cells in a low oxygen environment would have produced more ATP *via* oxidative phosphorylation than normoxic cells? The key to understanding this lies in the relationship between O_2_ and oxidative stress. Acute exposure to hypoxia increases ROS production, primarily through complex III (44–46). A fundamental role of HIF-1α may therefore be to serve as an “energy brake break”, protecting cells from oxidative stress by actively shutting down oxidative phosphorylation (1).

Zhang et al.’s findings were in contradiction to conventional wisdom at the time (which held that decreased oxidative phosphorylation in the setting of hypoxia was a result of inadequate oxygen supply) and deserve further investigation in other cell lines. We found that exposure of WT cells [in this case H9c2, albeit *via* a different cell type (myocardial)] to hypoxia leads to decreased ROS (1). We found that exposure of WT H9c2 cells to CoCl_2_ [a prolyl hydroxylase inhibitor that leads to H_2_O_2_ production and HIF-1α stabilization (45)] *increased* ROS in the setting of normoxia, similar to results reported by Chandel et al. (44). We also found that inhibition of HIF-1α in the setting of hypoxia leads to an *increase* in total ROS compared to WT cells exposed to hypoxia, confirming the essential role of HIF-1α in attenuating the production of ROS. In an earlier study by Zhang et al., HIF-1α knockouts produced more ROS than WT cells but during normoxia and hypoxia, in our study HIF-1α-inhibited cells produced more ROS than WT cells that were hypoxic, but less than normoxic cells.

We also observed that the specific Oxidative stress marker; total protein carbonyl content and Lipid peroxidation levels were markedly increased both in Hypoxia and in Cocl2 exposed cells compared to controls. Antioxidant (A2P) and HIF-1a inhibition (ECM) cells showed low Lipid peroxidation levels compared to Hypoxia and in Cocl2 exposed cells. Total Protein carbonyl (PC) content was also reduced with A2P treatment in Cocl2 exposed cells (Cocl2 + A2P), but these PC levels were not significantly changed in hypoxia + A2P and hypoxia + ECM exposed cells. The decreased anti-oxidant markers SOD and Catalase levels were significantly increased in A2P treated myocardium cells compared to Hypoxia and Cocl2 exposed cells. While, Hif1 inhibition (1%O2 + ECM) increased the cells total SOD levels compared to Hypoxia cells; however, Catalase levels were not significantly increased to control levels with HIF-1a inhibition (1%O2 + ECM). This difference can likely be attributed to differences in technique (knockout versus inhibition with ECM).

When we compare the normoxic to hypoxic WT H9c2 cells, we observed that HIF-1α and downstream HIF targets, VEGF, and EPO protein expression levels were increased, total ROS levels decrease, and oxidative stress (as measured by NADP/NADPH) decreases [though, specific oxidative markers; Protein carbonyl and Lipid Peroxidation levels increase in chemical hypoxia (Cocl2) and in low O2 (1% O2 hypoxia) but, these levels were less in 1%O2 cells (Cocl2 vs. 1% O2)]. This makes sense and is consistent with other published findings (1). With no interference on HIF-1α, hypoxia does lead to an overall decrease in ROS despite increasing superoxide production at Complex III (45). However, despite the clear increase in ROS levels when HIF-1α activity is inhibited by ECM in the setting of hypoxia (Figure 6), we found inconsistent changes in oxidative stress. Interestingly, our work suggests that ECM may impact HIF-1α expression itself. ECM is known to bind to the hypoxia response element and this is the primary mechanism by which it interacts with HIF-1α in cancer cell lines (47). However, some authors have demonstrated that ECM can lead to an *increase* in HIF-1α mRNA and protein expression during normoxic conditions (47). In H9c2 cells, we observed a decreased HIF-1α mRNA and protein expression in ECM exposed cells in the presence of hypoxia. This difference may be because the majority of research on ECM/HIF-1 has been focused on cancer cell lines [MCF-7 (human mammary tumor), U251 (human glioma), and HepG2 (hepatocellular carcinoma) cells] (47), which may behave differently than non-cancerous cardiomyocytes.

### Impact of Anti-oxidants on HIF-1α

While oxidative stress is undoubtedly harmful to organisms, increased ROS are also thought to be a necessary precursor for HIF-1α stabilization - mitochondrial ROS have been shown to stabilize HIF-1α (46, 48–50) and that ROS scavengers inhibit this process (44, 46, 49, 51). The interaction between ROS and HIF-1α has implications for both cancer (HIF-1α upregulation often confers a survival advantage to cancerous cells) and sepsis. The ability to decrease HIF-1 activity in cancerous cells may make these tissues more susceptible to radiation and chemotherapy (52, 53) (cancerous cells that tolerate hypoxia are more resistant to treatment, and HIF-1α up-regulation is a prerequisite to this hypoxia tolerance). Zhang et al. showed that HIF-1α was essential for resistant breast cancer tumors (54). Miles SL et al. showed that both ascorbic acid (AA) and ascorbate 2-phosphate (A2P) can antagonize HIF-1α protein stabilization in the setting of normoxia as well as during exposure to CoCl_2_ and prolyl hydroxylase (PHD) inhibition in melanoma cells (22). Because antioxidants reduce oxidative stress in non-cancerous cells, if they do prevent HIF-1α activity, this may ameliorate some of the benefits of attenuating oxidative stress. This may be why the Phase III trial of NOS inhibition in sepsis showed *increased* mortality in the treatment arm (55) despite many promising animal studies (56).

We found that the addition of the anti-oxidant A2P to normoxic H9c2 cells exposed to CoCl_2_ resulted in decreased ROS and HIF-1α expression. Similarly, the addition of A2P to hypoxic H9c2 cells also resulted in decreased HIF-1α expression, with no detectable change in ROS levels. Part of this discrepancy may be the relatively low ROS levels detected in hypoxia, as well as different sources of ROS – CoCl_2_ leads to peroxide overproduction, whereas hypoxia leads to superoxide production (45). Interestingly, the addition of A2P to H9c2 cells did not affect [Ca^2+^]_*i*_ in the setting of normoxia (with CoCl_2_) but significantly reduced [Ca^2+^]_*i*_ in the setting of hypoxia, which might be protective. Given the role of [Ca^2+^]_*i*_ in cell death as well as the possible role of moderate increases in [Ca^2+^]_*i*_ on preconditioning, further investigation seems warranted. We are not aware of any reports of anti-oxidants impacting [Ca^2+^]_*i*_ in the setting of hypoxia, although there is evidence that antioxidants improve skeletal function during exposure to hypoxia (57).

### HIF-1α and the Beclin-1/Autophagy Pathway

In the setting of hypoxia, we found that beclin-1 inhibition led to decreased ATG5 mRNA and protein expression, and we observed a significant decrease in autophagy, consistent with Zhang et al. Surprisingly, in the setting of normoxia, beclin-1 inhibition led to *increased* ATG5 mRNA and protein expression, and we observed a significant increase in autophagy. To determine whether mitochondrial autophagy does play a role in controlling ROS production, we measured total ROS levels in the setting of normoxia and hypoxia without and with beclin-1 inhibition and found that in both scenarios, beclin-1 inhibition leads to a substantial increase in total ROS levels. This strongly suggests that beclin-1 plays a prominent role in ROS attenuation independent of its role in mitochondrial autophagy. Most importantly, we found that while hypoxia leads to an increase in autophagy, which can be attenuated with the addition of antioxidants, HIF-1 inhibition does not affect this process at all despite the fact that beclin-1 reduces ATG5 mRNA and protein expression, suggesting that other non-HIF-1 factors drive the Beclin-1/autophagy pathway.

**FIGURE 12:**
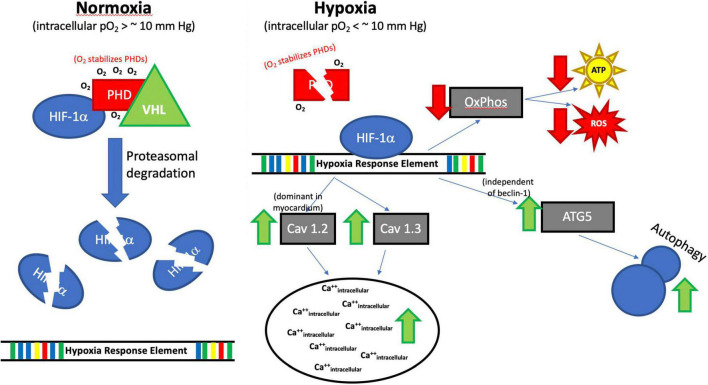
Proposed Impact of Hypoxia on HIF-1α. Although the full regulatory mechanism (Hypoxia→HIF-1α→Ca^++^/autophagy) still needs to be fully studied. Our results suggest that HIF-1α exerts several effects in H2c9 cells exposed to hypoxia, including upregulation of both Cav 1.2 and 1.3, which results in an increase in intracellular Ca^++^, increased ATG5 and autophagy through a beclin-1 independent mechanism, and reduced ATP and ROS production likely through a general slowdown in oxidative phosphorylation (OXPHOS). Prolyl hydroxylase domain protein (PHD), Von Hippel Lindau (VHL), Oxygen (O_2_), PO2 (partial pressure of oxygen), millimeters of mercury (mm Hg), Hif1α, Calcium channel, voltage-dependent, L type, alpha-1C subunit (Cav1.2), Calcium channel, voltage-dependent, L-type, alpha-1D subunit (Cav1.3), Adenosine triphosphate (ATP), Reactive oxygen species (ROS), ATG5 (Autophagy Related 5), Beclin-1, Autophagy Related (Becn1), ↑ increased, ↓ decreased.

Placing our study in the context of previously published work, our results suggest that HIF-1α exerts several effects in H2c9 cells exposed to hypoxia, including upregulation of both Cav 1.2 and 1.3, which results in an increase in intracellular Ca^++^. Increased ATG5 and autophagy through a beclin-1 independent mechanism and reduced ATP and ROS production likely through a general slowdown in oxidative phosphorylation (Figure 12). Echinomycin (ECM) appears to have a direct impact on HIF-1α expression in H2c9 cells and is not limited to its interaction with the hypoxia response element (HRE).

### Limitations

This study has several limitations. First, it was a study of cell lines, which means that the observed results are not directly applicable to intact organisms. Second, we used only one cell type (H9c2), which we selected because of the importance of the myocardium in long-term clinical outcomes in humans exposed to stress. Third, we only tested one anti-oxidant agent and one HIF-1α inhibitor. Though, we used pre-designed specificity-enhanced SignalSilence^®^ Beclin-1 siRNA II (reduces off-target effects), not using a separate control siRNA is possibly a limitation. The data are shown in Supplementary Figure 2 [the added immunofluorescence (IF) data in addition to the corresponding Cav (L-type of Ca2 + channel) protein expression levels and Cyto-ID Autophagy levels in cells]; Supplementary Figure 3 (Hypoxia/Oxidative stress ratio levels in cells) and the Supplementary Figure 4 [the compiled western blotting data of hypoxia, oxidative stress, and autophagy responsive proteins expression levels in Myocardium (H9C2 cells) are *N* = two experiment only, this is also a limitation because of lack of enough samples].

### Further Directions

Our results pose several questions that need answers through further work - how are antioxidants (e.g., A2P) able to exert an impact on HIF-1α protein expression without affecting detectable ROS levels? How ATG5 is upregulated despite the fact that beclin-1 is not upregulated in H9c2 cells exposed to hypoxia? How does beclin-1 mediate ROS production independent of autophagy? Involvement of PINK1/Parkin pathway in hypoxia-induced autophagy, studies with specific autophagy inhibitors (e.g., bafilomycin, chloroquine, etc.), in H9c2 cells. In addition, are moderate intracellular calcium increases during hypoxia exposure truly mediated by HIF-1α and upregulation of Cav 1.2 and if so, does this protect against cell death? What is the role of SERCA, PLN, Na + /Ca2 + exchangers, and plasma membrane Ca2 + ATPase on H9c2 cell’s intracellular Ca2 + levels in Hypoxia?

## Conclusion

Though additional confirmatory studies are needed, in the setting of hypoxia, HIF-1α upregulates both Cav 1.2 and 1.3 expression that results in an increase in intracellular Ca^++^, increases ATG5 and autophagy through a beclin-1 independent mechanism, and reduces ATP and ROS production in H2c9 cells.

## Data Availability Statement

The original contributions presented in this study are included in the article/Supplementary Material, further inquiries can be directed to the corresponding author.

## Author Contributions

HO: study design, the performance of the study, data analysis and interpretation, and manuscript writing and preparation. ML: data analysis and interpretation and manuscript writing. RT: design the study, data analysis and interpretation, and manuscript writing and preparation. All authors contributed to the article and approved the submitted version.

## Conflict of Interest

The authors declare that the research was conducted in the absence of any commercial or financial relationships that could be construed as a potential conflict of interest.

## Publisher’s Note

All claims expressed in this article are solely those of the authors and do not necessarily represent those of their affiliated organizations, or those of the publisher, the editors and the reviewers. Any product that may be evaluated in this article, or claim that may be made by its manufacturer, is not guaranteed or endorsed by the publisher.

## Supplementary Material

Supplementary Figure 1 **(A)** H9c2 cell viability was measured using the CellTiter-Glo^®^ Luminescent cell viability assay **(B)** Representative microscopy images at 20× magnification (scale bars 100 μm) showing H9c2 cells with Crystal violet staining. Data represents, *n* = 3 independent samples/group; Bars are mean ± S.D. Symbols *****, **+**, **#,** and **≠** denotes significant, “ns” represents not significant by two-way ANOVA- multiple comparisons test. P-values are *^ns^p* > 0.05, **p* < 0.05, ***p* < 0.01, ****p* < 0.001 and *****p* < 0.0001 compared to Control; ^+^*p* < 0.05, ^++^*p* < 0.01, ^+ + +^*p* < 0.001 and ^+ + + +^*p* < 0.0001 compared to Cocl2; ^#^*p* < 0.05, ^##^*p* < 0.01, ^###^*p* < 0.001 and ^####^*p* < 0.0001 compared to Hypoxia; ^≠^*p* < 0.05, ^≠^^≠^*p* < 0.01, ^≠^^≠^^≠^*p* < 0.001 and ^≠^^≠^^≠^^≠^*p* < 0.0001 compared to *si-becn1* (21%O2) Vs, *si-becn1* (1%O2).

Supplementary Figure 2 Representative images of Immunofluorescence (IF) staining showing Cav (L-type of Ca2 + channel) expression and Autophagy induction marker CYTO-ID levels. **(A,B)** IF staining showing Cav1.1 expression and Mean fluorescence intensity (MFI) levels. **(C,D)** IF staining showing Cav1.2 expression and MFI levels. **(E,F)** IF staining showing Cav1.3 expression and MFI levels. **(G,H)** Autophagy induction marker CYTO-ID staining showing autophagy and MFI levels. In the representative images green fluorescence (upper panel) showing the target protein expression, Hoechst (nuclei stain) in blue (center panel) and merge of both images (lower panel). Mean fluorescence intensity (MFI) levels were calculated using Fiji: an open-source platform for biological-image analysis. n = two experiments only, this data is an addition to the corresponding protein expression data shown (in the main text) with western blotting. IF images scale bar = 100 μm.

Supplementary Figure 3 Intracellular hypoxia/oxidative stress levels. The graph indicates **(A)** the relative Cellular Hypoxia/Oxidative stress levels (expressed ROS and Hypoxia fluorescent levels were normalized with nuclei stain Hoechst 33342) in H9c2 cells treated with ECM, A2P, siRNA-Beclin1, and grown under CoCl2, Hypoxia (1%O2) and Normoxia conditions (21%O2). **(B)** Immunofluorescence (IF) images of ROS-ID^®^ Hypoxia/Oxidative stress detection staining showing ROS expression in green (*panel 1 from top*) Hypoxia in Red (*panel 2*), nuclei stain in blue (*panel 3*) and merge of both images (*lower panel 4*). Hypoxia and ROS levels were increased in Cocl2, 1%O2, exposed cells compared to control cells grown under normoxia. A2P and ECM treated cells’ Hypoxia levels were decreased compared to Cocl2 and Hypoxia groups. SiRNA-Beclin1 inhibition also expressed low hypoxia levels. IF images scale bar = 100 μm. Data represents, *n* = 2 experiments only, this Hypoxia/Oxidative stress levels data in addition to the data shown in Figures 8 (Hif1α expression indicates-Hypoxia) and Figure 6 (Total ROS levels).

Supplementary Figure 4 **(A)** The compiled western blotting data of several Hypoxia, Oxidative stress, and Autophagy responsive proteins expression levels in Myocardium (H9C2 cells), **(B)** represents normalized density levels protein expression patterns in response to hypoxia-mimetic agent cobalt chloride (CoCl2), low oxygen (1%o2) hypoxia, Echniomycin (HIF inhibitor), A2P (anti-oxidant), and beclin-1 inhibition. Autophagy Related 12 (ATG12), Autophagy Related 16 like (ATG16L), Superoxide Dismutase 1 (SOD1), Heme Oxygenase 1 (HO-1), Nitric Oxide Synthase, Inducible (iNOS). BCL2/Adenovirus E1B 19 KDa Protein-Interacting Protein 3 (Bnip3), BCL2/Adenovirus E1B 19 KDa Protein-Interacting Protein 3-Like (Bnip3l), Bcl-2 (B-cell lymphoma 2), Beta-actin (b-act), Beclin1/Becn1 (Coiled-Coil, Moesin-Like BCL2 Interacting Protein). Hypoxia-inducible factor 2-alpha (Hif2a), Hypoxia-inducible factor 1-beta (Hif2b), Mechanistic Target of Rapamycin Kinase (mTOR), AKT Serine/Threonine Kinase (AKT), Protein Kinase AMP-Activated Catalytic Subunit (AMPK). For some of the proteins western blotting data shown here, we do not have enough number “n” to see the expression differences between the groups using Statistical Comparisons. Hence, we are submitting this data only as a supplementary to show the expression levels of Hypoxia response protein levels in H9c2 cells.
