# Supplementary figures and images for: Molecular and Cellular Response of the Myocardium (H9C2 Cells) Towards Hypoxia and HIF-1α Inhibition
Source: Front Cardiovasc Med. 2022 Jul 19;9:711421. doi: 10.3389/fcvm.2022.711421 (PMC9343679; doi:10.3389/fcvm.2022.711421)

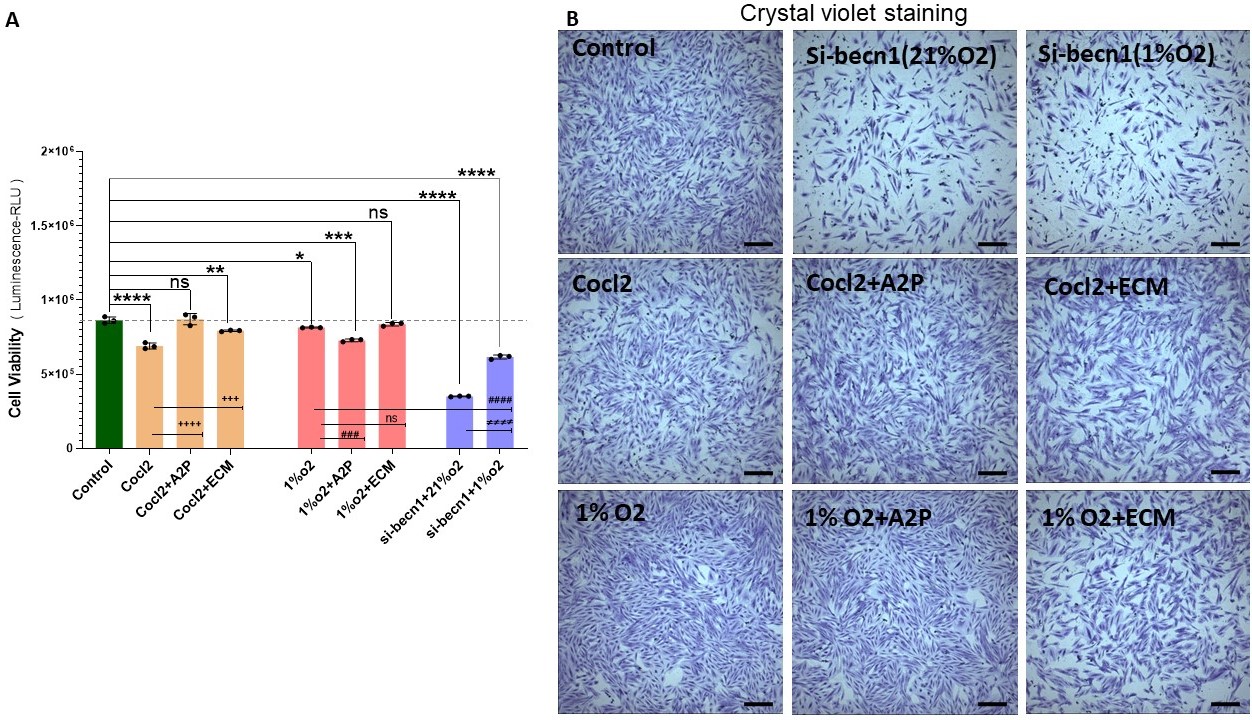

Supplement: Supplementary Figure 1 — (A) H9c2 cell viability was measured using the CellTiter-Glo® Luminescent cell viability assay (B) Representative microscopy images at 20× magnification (scale bars 100 μm) showing H9c2 cells with Crystal violet staining. Data represents, n = 3 independent samples/group; Bars are mean ± S.D. Symbols *, +, #, and ≠ denotes significant, “ns” represents not significant by two-way ANOVA- multiple comparisons test. P-values are nsp > 0.05, *p < 0.05, **p < 0.01, ***p < 0.001 and ****p < 0.0001 compared to Control; +p < 0.05, ++p < 0.01, + + +p < 0.001 and + + + +p < 0.0001 compared to Cocl2; #p < 0.05, ##p < 0.01, ###p < 0.001 and ####p < 0.0001 compared to Hypoxia; ≠p < 0.05, ≠ ≠p < 0.01, ≠ ≠ ≠p < 0.001 and ≠ ≠ ≠ ≠p < 0.0001 compared to si-becn1 (21%O2) Vs, si-becn1 (1%O2). [file Image_1.JPEG]

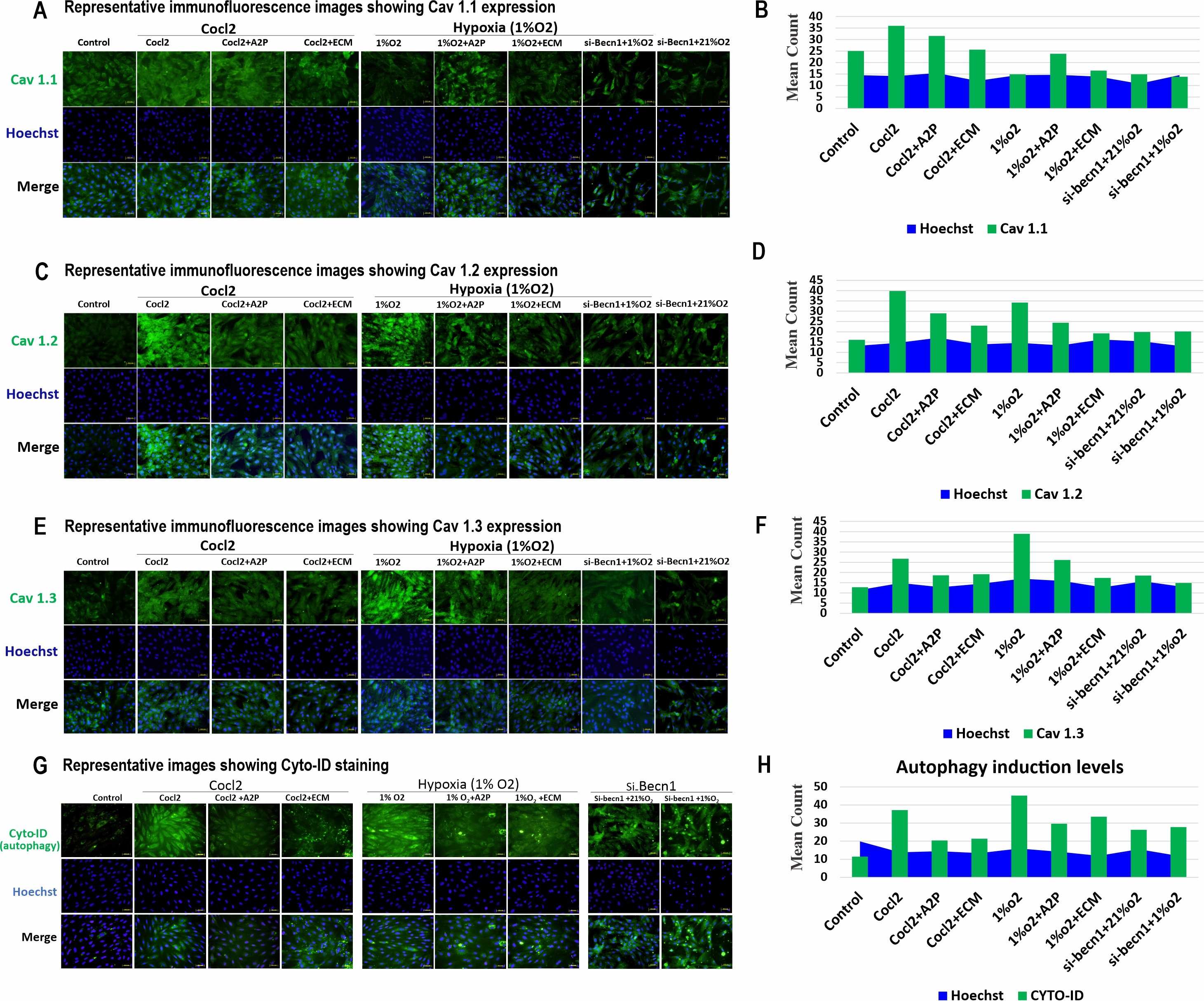

Supplement: Supplementary Figure 2 — Representative images of Immunofluorescence (IF) staining showing Cav (L-type of Ca2 + channel) expression and Autophagy induction marker CYTO-ID levels. (A,B) IF staining showing Cav1.1 expression and Mean fluorescence intensity (MFI) levels. (C,D) IF staining showing Cav1.2 expression and MFI levels. (E,F) IF staining showing Cav1.3 expression and MFI levels. (G,H) Autophagy induction marker CYTO-ID staining showing autophagy and MFI levels. In the representative images green fluorescence (upper panel) showing the target protein expression, Hoechst (nuclei stain) in blue (center panel) and merge of both images (lower panel). Mean fluorescence intensity (MFI) levels were calculated using Fiji: an open-source platform for biological-image analysis. n = two experiments only, this data is an addition to the corresponding protein expression data shown (in the main text) with western blotting. IF images scale bar = 100 μm. [file Image_2.JPEG]

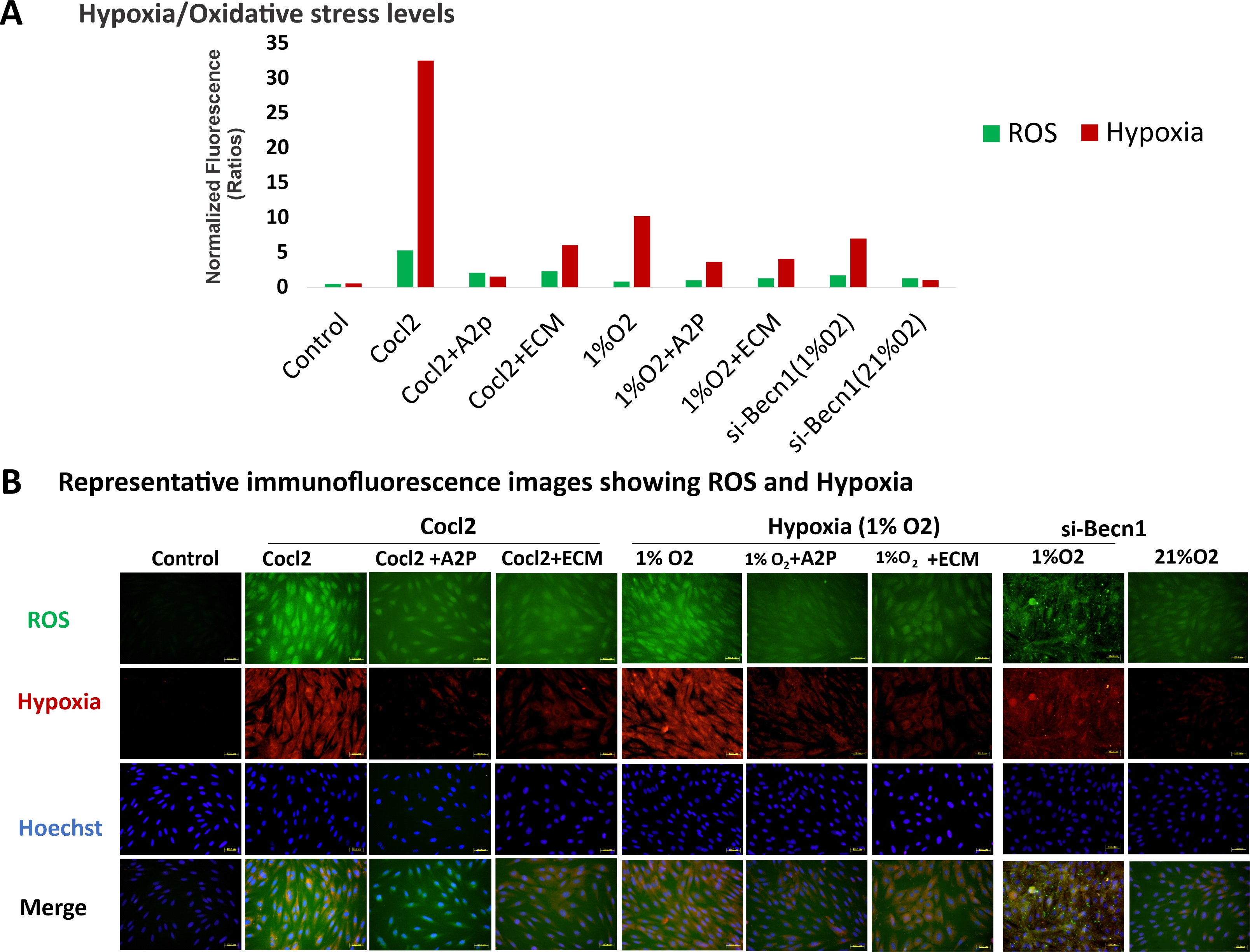

Supplement: Supplementary Figure 3 — Intracellular hypoxia/oxidative stress levels. The graph indicates (A) the relative Cellular Hypoxia/Oxidative stress levels (expressed ROS and Hypoxia fluorescent levels were normalized with nuclei stain Hoechst 33342) in H9c2 cells treated with ECM, A2P, siRNA-Beclin1, and grown under CoCl2, Hypoxia (1%O2) and Normoxia conditions (21%O2). (B) Immunofluorescence (IF) images of ROS-ID® Hypoxia/Oxidative stress detection staining showing ROS expression in green (panel 1 from top) Hypoxia in Red (panel 2), nuclei stain in blue (panel 3) and merge of both images (lower panel 4). Hypoxia and ROS levels were increased in Cocl2, 1%O2, exposed cells compared to control cells grown under normoxia. A2P and ECM treated cells’ Hypoxia levels were decreased compared to Cocl2 and Hypoxia groups. SiRNA-Beclin1 inhibition also expressed low hypoxia levels. IF images scale bar = 100 μm. Data represents, n = 2 experiments only, this Hypoxia/Oxidative stress levels data in addition to the data shown in Figures 8 (Hif1α expression indicates-Hypoxia) and Figure 6 (Total ROS levels). [file Image_3.JPEG]

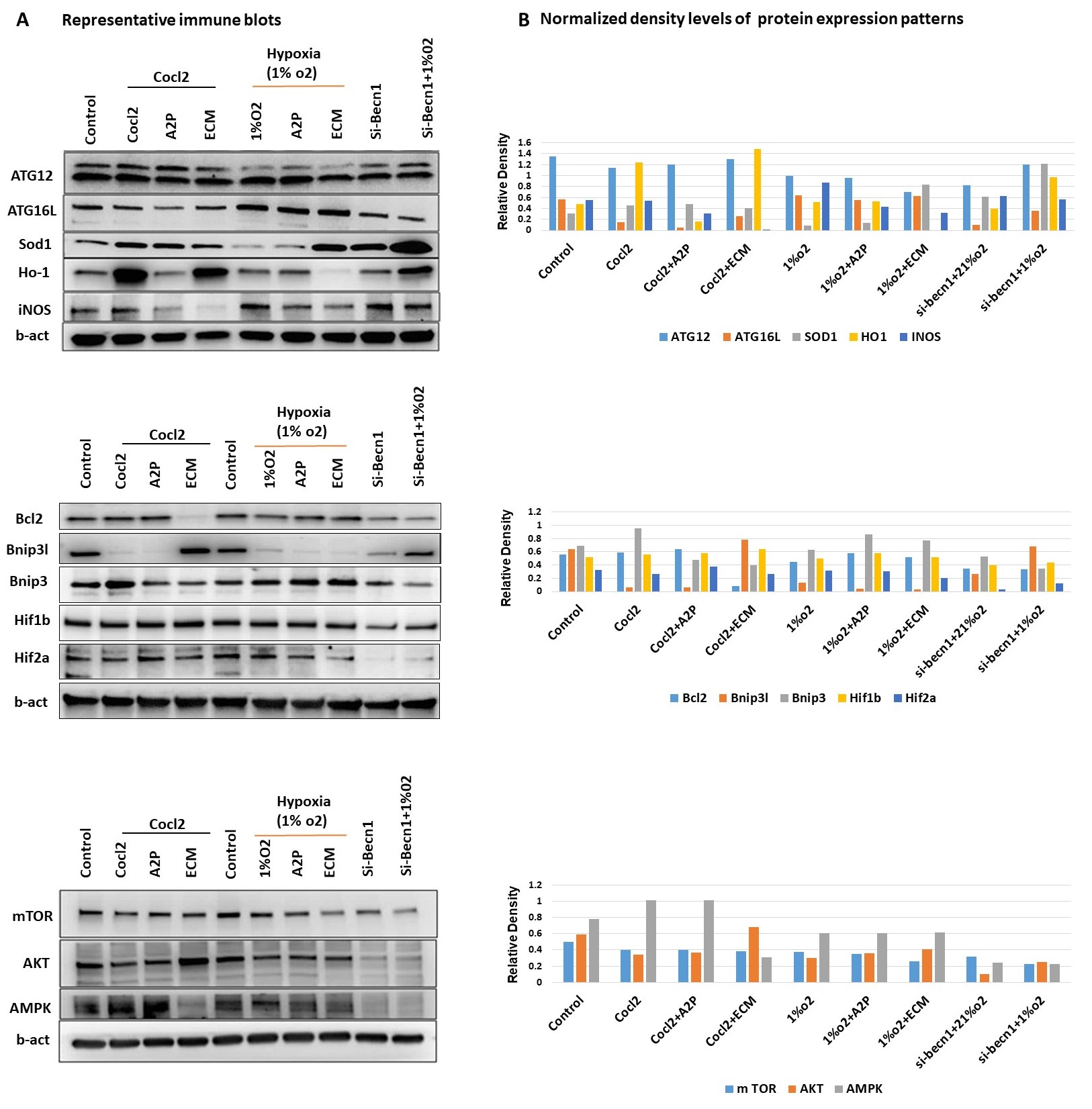

Supplement: Supplementary Figure 4 — (A) The compiled western blotting data of several Hypoxia, Oxidative stress, and Autophagy responsive proteins expression levels in Myocardium (H9C2 cells), (B) represents normalized density levels protein expression patterns in response to hypoxia-mimetic agent cobalt chloride (CoCl2), low oxygen (1%o2) hypoxia, Echniomycin (HIF inhibitor), A2P (anti-oxidant), and beclin-1 inhibition. Autophagy Related 12 (ATG12), Autophagy Related 16 like (ATG16L), Superoxide Dismutase 1 (SOD1), Heme Oxygenase 1 (HO-1), Nitric Oxide Synthase, Inducible (iNOS). BCL2/Adenovirus E1B 19 KDa Protein-Interacting Protein 3 (Bnip3), BCL2/Adenovirus E1B 19 KDa Protein-Interacting Protein 3-Like (Bnip3l), Bcl-2 (B-cell lymphoma 2), Beta-actin (b-act), Beclin1/Becn1 (Coiled-Coil, Moesin-Like BCL2 Interacting Protein). Hypoxia-inducible factor 2-alpha (Hif2a), Hypoxia-inducible factor 1-beta (Hif2b), Mechanistic Target of Rapamycin Kinase (mTOR), AKT Serine/Threonine Kinase (AKT), Protein Kinase AMP-Activated Catalytic Subunit (AMPK). For some of the proteins western blotting data shown here, we do not have enough number “n” to see the expression differences between the groups using Statistical Comparisons. Hence, we are submitting this data only as a supplementary to show the expression levels of Hypoxia response protein levels in H9c2 cells. [file Image_4.JPEG]
