# Supplementary material for: Molecular and Cellular Response of the Myocardium (H9C2 Cells) Towards Hypoxia and HIF-1α Inhibition
Source: Front Cardiovasc Med. 2022 Jul 19;9:711421. doi: 10.3389/fcvm.2022.711421 (PMC9343679; doi:10.3389/fcvm.2022.711421)
Supplement: Supplementary file 5 [file Table_1.docx]

**Supplemental Table 1: Quantitative PCR** Primers used for Mitochondrial DNA and target mRNA levels quantification. All primers were purchased from Sigma and Invitrogen, USA.

| **Gene** | **Forward (5'-3')** | **Reverse (5'-3')** |
| --- | --- | --- |
| *Hif1a* | GAAAGGATTACTGAGTTGATGG | CAGACATATCCACCTCTTTTTG |
| *ATG5* | CTGCAGAATGATTTGACCAG | AACAGCTTCTGAATGAAAGG |
| *Becn1* | GAAATTTCAGAGGTACCGAC | GCAACTCCTTAGATTTGTCTG |
| *Cav1.1* | AAAAAGATCTGGAAGAGAACC | ACCCATAATATTCCTCCTGG |
| *Cav1.2* | TTAAAAGTGATTGCCTACGG | CACTAAAAAGCCCTACAACC |
| *Cav1.3* | CTCAGATATAGTAGCTGAAGAGG | GTCAAAATTAGTGATGCCTCC |
| *Hprt1* | ACTGGTAAAACAATGCAGAC | CCTGAAGTGCTCATTATAGTC |
| *Tuba1a* | ATTATGAGGAGGTTGGTGTG | TGTTGGACCAGAATAAACATG |
| *Mt_Nd1* | GCAGGACCATTCGCCCTATT | GGGGTAGGATGCTCGGATTC |
| *Mt_Nd6* | CGCAAACAATGACCACCCAG | CCCGGAGACTTGAGGGTCTA |
